# Neoadjuvant leukocyte interleukin injection immunotherapy improves overall survival in low-risk locally advanced head and neck squamous cell carcinoma –the *IT-MATTERS* study

**DOI:** 10.3389/pore.2025.1612084

**Published:** 2025-03-21

**Authors:** Eyal Talor, József Tímár, Philip Lavin, John Cipriano, Dusan Markovic, Andrea Ladányi, Andrey Karpenko, Igor Bondarenko, Srboljub Stosic, Hrvoje Sobat, Aliaksandr Zhukavets, Nazim Imamovic, Chih-Yen Chien, Magdalena Bankowska-Wozniak, Mihály Kisely, Rajko Jovic, James Edward Massey Young, Sheng-Po Hao

**Affiliations:** ^1^ CEL-SCI Corporation, Vienna, VA, United States; ^2^ Department of Pathology, Forensic and Insurance Medicine Semmelweis University, Budapest, Hungary; ^3^ Boston Biostatistics Research Foundation, Framingham, MA, United States; ^4^ Ergomed Group, Guildford, United Kingdom; ^5^ Department of Surgical and Molecular Pathology and National Tumor Biology Laboratory, National Institute of Oncology, Budapest, Hungary; ^6^ Leningrad Regional Oncology Dispensary, Saint Petersburg, Russia; ^7^ Municipal Non-Commercial Enterprise “City Clinical Hospital No. 4” of Dnipro City Council, Head and Neck Pathology Department, State Institution “Dnipropetrovsk Medical Academy Under the Ministry of Health of Ukraine”, Oncology and Medical Radiology, Dnipro, Ukraine; ^8^ Military Medical Academy, Clinic for Maxillofacial Surgery, Belgrade, Serbia; ^9^ University Clinical Hospital Center “Sestre Milosrdnice”, University Hospital for Tumors, Zagreb, Croatia; ^10^ Department of Surgery, N.N.Alexandrov National Cancer Centre of Belarus, Minsk, Belarus; ^11^ Clinic for Plastic and Maxillofacial Surgery, University Clinical Center Tuzla, Tuzla, Bosnia and Herzegovina; ^12^ Department of Otolaryngology, Kaohsiung Chang Gung Memorial Hospital and Chang Gung University College of Medicine, Kaohsiung, Taiwan; ^13^ Department of Surgery, Centrum Onkologii im Prof. F. Lukaszcsyka, Bydgoszcz, Poland; ^14^ Department of Surgery, Markusovszky Lajos Teaching Hospital, Szombathely, Hungary; ^15^ Department of Laryngology, Oncology and Phoniatrics, Clinic for Otorhinolaryngology and Head and Neck Surgery, University Clinical Centre of Vojvodina, Novi Sad, Serbia; ^16^ St Joseph Healthcare Hamilton, McMaster University, Hamilton, ON, Canada; ^17^ Department on Head and Neck Surgery, Shin Kong Wu Ho-Su Memorial Hospital, Taipei, Taiwan

**Keywords:** Leukocyte interleukin injection (LI), immunotherapy, neoadjuvant, SCCHN, locally advanced disease, low-risk for recurrence, lower disease burden

## Abstract

The randomized controlled pivotal phase 3 study evaluated efficacy and safety of neoadjuvant complex biologic, Leukocyte Interleukin Injection (LI), administered for 3 consecutive weeks pre-surgery, in treatment naïve resectable locally advanced primary squamous cell carcinoma of oral cavity and soft palate. Randomization 3:1:3 to LI+/-CIZ (cyclophosphamide, indomethacin, and zinc)+SOC, or SOC (standard of care) alone. LI-treated patients received 400 IU (as interleukin-2 equivalent; 200 IU peritumorally, 200 IU perilymphatically) sequentially, daily 5 days/week for 3 weeks before surgery. All subjects were to receive SOC. Post-surgery, patients with low risk for recurrence were to receive radiotherapy, while those with high risk received concurrent chemoradiotherapy. Median follow-up was 56 months. There were 923 ITT (Intent-to-Treat) subjects (380 ITT low-risk and 467 ITT high-risk). Pre-surgery objective early response (45 objective early responders; 5 complete responses [CRs], 40 partial responses [PRs], confirmed by pathology at surgery. LI (+/− CIZ) had 8.5% objective early responders (45/529 ITT) and 16% objective early responders (34/212 ITT low-risk) vs. no reported SOC objective early responders (0/394 ITT). Objective early responders significantly lowered death rate to 22.2% (ITT LI-treated), 12.5% (ITT low-risk LI + CIZ + SOC), while the ITT low-risk SOC death rate was 48.7%. Thus, objective early response impacted overall survival (OS); proportional hazard ratios were 0.348 (95% CI: 0.152–0.801) for ITT low-risk LI-treated, 0.246 (95% CI: 0.077–0.787) for ITT low-risk LI + CIZ + SOC. ITT low-risk LI + CIZ + SOC demonstrated significant OS advantage vs. ITT low-risk SOC (unstratified log-rank p = 0.048; Cox hazard ratio = 0.68; 95% CI: 0.48–0.95, Wald p = 0.024 [controlling for tumor stage, tumor location, and geographic region]). Absolute OS advantage increased over time for ITT low-risk (LI + CIZ + SOC)-treated vs. ITT low-risk SOC: reaching 14.1% (62.7% vs. 48.6%) at 60 months, with 46.5 months median OS advantage (101.7 months vs. 55.2 months), respectively. Quality of life benefit for complete responders sustained for >3 years post LI treatment. Percent treatment-emergent adverse events were comparable among all treated groups. No excess safety issues were reported for LI over SOC alone post-surgery. NCT01265849, EUDRA:2010-019952-35.

## Introduction

SCCHN is the sixth most common cancer worldwide, with approximately 890,000 cases annually, and 460,000 deaths [1]. In the United States ∼71,000 cases and ∼16,000 deaths occur annually [2]. Approximately two-thirds of all SCCHN cases present at diagnosis with advanced primary disease [3]. Based on the National Comprehensive Cancer Network (NCCN) Guidelines [3], we estimate that about 40% of the advanced primary SCCHN patients fall within the low risk (LR) for recurrence category. Current standard of care (SOC) for locally advanced (LA) primary oral squamous cell carcinoma (OSCC) is surgery followed by radiotherapy (RTx) for LR or concurrent chemoradiotherapy (CRTx) with cisplatin (100 mg/m^2^) for high risk (HR) for recurrence, exhibiting pathologic criteria (collectively referred to as “adverse pathologic features,” per the NCCN Guidelines recommendations) determined at surgery [3]. Though SOC improved over time, the 5-year overall survival (OS) for the LA resectable OSCC remains below 50% [4]. Therefore, a large, underserved proportion of OSCC patients have not benefited from existing SOC. Furthermore, the current SOC imparts a high treatment burden [5, 6]. Consequently, these patients have an unmet medical need with no advances in OS in decades.

Immune checkpoint inhibitors (ICIs) and anti-epidermal growth factor receptor antibody are other novel therapies approved for SCCHN, but these therapies are only for treating tumors that are non-resectable, have recurred, or metastasized [7].

Neoadjuvant immunotherapy maintains the physiological connection between primary tumor and the draining lymph nodes essential for antitumor immune responses. The enhanced local antitumor immune responses may result in clinical to pathological downstaging, improve the efficacy of surgical resection. Furthermore, the systemic effects of neoadjuvant immunotherapy can be crucial from the point of systemic antitumor immune response important to fight circulating tumor cells. Neoadjuvant immunotherapy is now part of the protocols of triple-negative breast cancer and NSCLC and many phase 3 trials are running in other cancer types [8, 9].

In SCCHN several neoadjuvant trials have tested the potential efficacy of anti-PD-1/PD-L1 and or anti-CTLA-4 antibodies, resulting in downstaging in 19%–100% [10–12]. On the other hand, several trials looked for efficacy of immunotherapy combined with chemo- or radiotherapy in neoadjuvant setting, resulting in increased pathological complete response rate [11, 12]. In addition, the current adjuvant treatment options including immunotherapy do not result in improved outcomes for the majority of non-SOC-responding patients, partly due to multi-resistant tumor cells and to replacements of the tumor cell clones in case of disease recurrence [4, 7]. Therefore, a neoadjuvant immune-based treatment approach is becoming more accepted and may be more advantageous in head and neck oncology.

Cytokine therapy was shown to work in early studies using intratumoral and peritumoral immunotherapy in the neoadjuvant setting [13–17]. More recently IRX-2, a natural cytokine mixture was tested in neoadjuvant setting as monotherapy in stage II-IV OSCC [18] or as combinational therapy with anti-PD-L1 (durvalumab) [19] for the treatment of metastatic or recurrent SCCHN, with limited success.

Leukocyte Interleukin Injection (LI; also called Multikine) is a natural cytokine mixture [20–22]. Its anti-tumor activity is associated with inflammatory response localized to the tumor mass [21–24] and shown to overcome tumor suppression of host anti-tumor response [21–23]. The mechanism of action of LI points to augmenting immune activation [21]. Phases 1 and 2 data support this hypothesis [21–23] showing LI augmentation of CD4^+^ T (and others) cell infiltrates and CD4/CD8 ratio in the tumor and tumor microenvironment (TME) [21–23]. TME changes were observed in patients receiving LI + CIZ (cyclophosphamide, indomethacin, and zinc) prior to SOC [21–23]. More importantly, a relatively high response rate of 42% has been achieved with this novel type of immunotherapy [22]. No LI safety issues were noted in Phase 1 and 2 studies, exceedingly important in light of current SOC’s high treatment burden [5]. We hypothesized that pro-inflammatory cytokines (i.e., LI) could overcome local immune suppression by the tumor, break tumor tolerance, allowing for effective local anti-tumor immune response, resulting in increased OS [22, 25]. The clinical benefit of LI neoadjuvant immunotherapy, a pioneering treatment for newly diagnosed treatment-naïve locally advanced resectable primary OSCC and soft palate slated for treatment with “intent to cure” (current SOC) was evaluated as the first treatment following diagnosis in the randomized, controlled, multi-center phase 3 (IT-MATTERS) study. The proposed indication of LI is for node negative LA primary treatment naïve, resectable OSCC + soft-palate presumed at diagnosis, consistent with LR. PET-CT/MRI would allow diagnosis/screening of acceptable patients for LI treatment.

The treatment of locally advanced SCCHN requires complex multi-dimensional pre-operative planning leaving a limited time-window to test novel neoadjuvant treatments. Pre-operative neoadjuvant administration may provide guidance on biological markers for early patient identification/selection in future clinical investigations, improving personalized medicine.

## Materials and methods

### Study design

**FIGURE 1:**
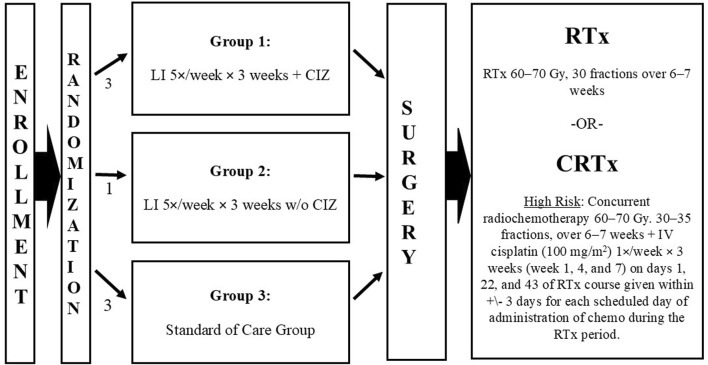
Study schema from enrollment through disease-directed therapy. Abbreviations: CIZ, cyclophosphamide, indomethacin, zinc; CRTx, chemoradiotherapy; IV, intravenous; po, orally; RTx, radiotherapy; TID, three times daily.

This event-driven study concluded when 298 deaths were documented in the two comparator arms (LI + CIZ + SOC and SOC alone) (Figure 1). Objective early response (OER) was determined by RECIST v1.0 and confirmed by pathology at surgery. Subjects were followed a minimum of 48 months after completing study drug and SOC-related procedures (median follow-up 56 months). Follow-up timing was modeled after the NCCN Guideline recommendation assessed safety, OS, and recurrence of disease (including local regional control [LRC]) until death, lost to follow-up or withdrawn consent. Prolonged follow-up was necessary to reach the targeted 298 deaths (events) in the two study comparator arms (Groups 1 and 3) for the evaluable ITT population.

All study drugs were sourced centrally and distributed to all sites globally. Site investigator training was provided via multiple study investigator meetings.

The study was approved by site Institutional Review Boards and/or Central Ethics Committee (depending on the institution and country requirements) and was conducted in accordance with the Declaration of Helsinki, Good Clinical Practice guidelines, and met all International Council for Harmonization (ICH) Good Clinical Practice requirements, including the European Union General Data Protection Regulation. Written informed consent (in local languages) was obtained from all patients. All patients volunteered; compensation was not offered to participants. ClinicalTrials.gov identifier: NCT01265849, EudraCT identifier: 2010-019952-35.

The study was launched in December 2010 and followed NCCN Guidelines treatment for locally advanced OSCC/soft palate (Figure 1), last patient entered September 2016, follow-up ended December 2020. The NCCN risk groups (low risk and high risk for recurrence) are only determined following surgical resection (tumor/lymph nodes) and risk is a known major prognostic factor for survival. Therefore, patients could not be stratified for randomization, by risk. To compensate, and prospectively assess patients by risk, the sample size was made large enough (>80% power) for each independent Intention-to-Treat (ITT) risk population (low risk (LR) and high risk (HR) for recurrence) to detect a 0.647 OS hazard ratio for LI + CIZ + SOC over SOC alone.

A prospectively determined analysis by risk in the protocol, elaborated in the study Statistical Analysis Plan including shell tables (finalized/signed prior to study database lock) allowed for both analysis by risk, and to draw risk-based conclusions.

### Patients

Eligibility criteria included age ≥18 years, Karnofsky score >70/100, life expectancy >6 months, able and willing to provide written informed consent; LA treatment naïve resectable tumors in the oral cavity or soft palate, adequate bone marrow function, normal levels of bilirubin and creatinine, no prior therapy with interleukin-2, interleukin-1 or other biological response modifiers, or treated with immune suppressive drugs, or anti-cancer agents, in the past year. Exclusions included congestive heart failure, active/bleeding peptic ulcer, acute viral or bacterial infection, abnormal cellular immunity, on hemo/peritoneal dialysis, tumors in other anatomical locations of the head and neck.

### Randomization and masking

Eligible subjects were randomized 3:1:3 to one of three treatment regimens [LI + CIZ + SOC (Group 1); LI + SOC (Group 2); or SOC alone control (Group 3) – had no treatment prior to surgery]. Subjects were stratified by tumor stage and location (Figure 1) within each geographical region. The randomization schema did not show significant differences in the treated groups, as a result the randomization stratification was equally represented in each treatment group in both treated and control subjects within each of the region’s sites and the study as a whole (NCT01265849). The study was conducted as open label since the primary endpoint was OS; the sponsor was blinded to all study data from fall 2016 to data reveal in mid-June 2021 (data lock occurred December 2020).

### Investigational drug

LI is a non-autologous, cell-free immunotherapeutic complex biological mixture containing pro-inflammatory cytokines, other human cytokines/lymphokines, produced in a validated Good Manufacturing Practice aseptic process from *in vitro* culture of peripheral blood mononuclear cells (PBMCs), which are isolated from Source Leukocytes (a Food and Drug Administration-licensed product for the further manufacture of biologics) [20]. The Source Leukocytes are prepared from blood donations obtained from Normal Blood Donor population in the United States, in a US FDA licensed facilities authorized to collect blood for transfusion. All blood donors must not be in any deferral category at the current or past blood donation. All donors must test negative to all US FDA mandated test panel for viruses and other advantageous agents. The cell-free culture supernatant undergoes clarification, purification, virus removal, and formulated to specifications before aseptic fill/finish. The injectable product has a 2-year shelf-life at −20°C. The biological activity equivalent of LI is determined by comparing the biological activity of IL-2 present in LI against the Second WHO International Standard 86/500 for IL-2 using a validated cytotoxic T-lymphoid line (CTLL), which growth and proliferation is dependent on IL-2, by a radiothymidine incorporation end point (the CTLL-2 bioassay). The in-house validated CTLL-2 Bioassay was adapted from Watson et al [26] and standardized against the 2nd International Standard for IL-2 [27]. The LI finished drug product therefore derives its units from the International Standard for IL-2.

### Procedures

Subjects randomized to Groups 1 and 2 received LI, 2 mL daily total 400 IU as interleukin-2 equivalent, half-daily dose (1 mL) peritumorally and half-daily dose (1 mL) perilymphatically (5 times per week for 3 consecutive weeks). Perilymphatic administration was performed near the draining lymphatic chain ipsilateral to the oral cavity tumor location into the sternocleidomastoid muscle about 1.5 cm below the muscle insertion point. Group 1 also received CIZ (referred to as LI + CIZ treatment) prior to receiving SOC, while Group 3 received only SOC.

CIZ (cyclophosphamide, indomethacin, and zinc): CIZ (cyclophosphamide one-time only 300 mg/m^2^ (i.v., bolus) 3 days prior to the first LI administration; indomethacin 25 mg po tid; zinc 15–45 mg zinc as multivitamins po, once daily, both indomethacin and zinc-multivitamins daily from the first day of LI administration to 1 day prior to surgery) is given to augment LI’s activity.

SOC: all subjects received the current SOC, which includes surgery (complete surgical resection of primary tumor and any positive lymph nodes) followed by either radiotherapy (mandatory) or concurrent chemoradiotherapy (for subjects that are determined to be at high risk post-surgery according to the NCCN guidelines). Radiotherapy was given per protocol at a total of ≥60 Gy to ≤70 Gy (in 30–35 fractions over a 6- to 7-week period).

### Histopathology

Tumor samples (pre- or post-treatment/surgical resections) as well as neck dissections have been processed for histopathology examination according to the actual (2010) guidelines of College of American Pathologists and the Royal College of Pathologists using standard formalin fixation/paraffin embedding (FFPE) procedures. To standardize reporting, uniform case report sheets were used containing predefined checklists (*Pathology protocol 1–4,* Supplementary Material). Pathological staging was done according to the AJCC/UICC TNM 7th edition. Case report sheets of all randomized patients have been sent to the Central Pathology Laboratory which evaluated those and in case of any non-adherence to protocols corrections have been performed.

High-risk subjects were defined as those with: positive surgical margins, two or more clinically positive nodes, or extracapsular nodal spread (any or all of the above) and as recommended by the National Comprehensive Cancer Network Guidelines.

### Outcome

Primary efficacy endpoint was OS in the ITT population, analyzed by unstratified log rank test. The statistical analysis plan prospectively specified analyses by risk for LR and HR (risk is determined post-surgery as defined per NCCN). Safety was evaluated for all randomized subjects from signed informed consent to study end, death, or withdrawn consent. Subjects were analyzed for safety as treated.

### Response evaluation

The OER to neoadjuvant LI was assessed using RECIST v1.0 and all CRs/PRs were confirmed at surgery by pathology. Response outcome was analyzed for study treatment regimen, risk, and baseline disease stage and presented for overall and pre-defined LR. The study subjects also had pre-surgical tumor responses in the form of downstaging. Tumor-lymph node (TN) scores were assessed per protocol at screening and at (prior to) surgery. To standardize these scores to the downstage migration reported in the literature [28], we retrospectively mapped these TN scores to AJCC Stage using the AJCC TNM Cancer Staging Manual, 7th edition. Changes from screening to surgery were classified as downstage (PSD), upstage, or no change, as follows: “Downstage”: improvement in T score and/or N score without either worsening T score or N score (e.g., T2N1 to T1N0 or to T1N1); “Upstage”: worsening in T score and/or N core without either improving T score or N score (e.g., T2N1 to T3N1 or T2N2); and “No change”: neither downstage nor upstage. The time to death was further evaluated using separate proportional hazard models to estimate the hazard ratio and corresponding two-sided hazard ratio as explained by OER and AJCC stage migration from baseline.

### Safety and quality of life evaluation

Safety was assessed/reported by investigators using common toxicity criteria version 5, Medical Dictionary for Regulatory Activities (MedDRA) version 23.0; Treatment-Emergent Adverse Events (TEAEs) were classified using MedDRA System Organ Class and Preferred Terms as well as relatedness to the investigational product consistent with ICH Good Clinical Practice. Adverse events (AEs) were evaluated pre-surgery, during subsequent disease-directed therapy (DDT), post-DDT, and overall (entry to exit).

Validated Quality of Life (QoL) instruments (EORTC QLQ-C30 and EORTC QLQ-H&N 35) [29, 30] were used across all sites.

### Statistical analysis

The study was hypothesis-driven for primary and secondary efficacy measures. Originally powered at 80% to detect a 3-year 10% absolute advantage of LI-treated over control, 784 cases were required. The final sample size (ITT, n = 923) permitted the retrospective power to be calculated separately for the LR and HR populations. Given the longer accrual (5 years) with longer follow-up (an additional 4 years), the retrospective power was 88% for the HR group (n = 467) and 81% for the LR group (n = 380) to detect a 0.647 hazard ratio for the survival advantage of LI-treated vs. control (SOC); this could not be computed at study launch since no supporting data existed for that purpose. A two-sided <0.05 p-value was considered statistically significant.

Prospectively defined efficacy analyses were assessed by study intervals; the interval from randomization to last follow-up/death was primary for OS, PFS, and LRC, while randomization to surgery was the primary interval for OER. Cox proportional hazard models were pre-defined for OS, PFS, LRC with disease site, stage, and geographical region as covariates in addition to treatment; contrast tests were used for pairwise comparisons of Groups 1 (primary), Group 2 (secondary) vs. Group 3 control, for simultaneous comparisons without penalty.

## Results

### Study population

The study enrolled 928 randomized patients; follow-up continued to database lock. Twenty countries (3 continents) participated. The study screened 1,236 subjects, randomized and treated 928 at 78 sites. Five randomized subjects were excluded, 4 cases due to the 2014 Crimean war could not be followed (hospital destruction), 1 United States case (Principal Investigator [PI] did not sign the full Case Report Form), leaving 923/928 (99.5%) ITT subjects for analysis. There were no reported deaths due to COVID-19; the pandemic did not impact the study.

Table 1 displays the 923 ITT subjects: LI + CIZ + SOC (n = 395; Group 1), LI + SOC (n = 134; Group 2), and SOC alone (n = 394; Group 3). A total of 92.2% (851/923) subjects underwent surgery with median/mean times to surgery 35/36 days for Group 1 and 2 in contrast to 12/13 days for Group 3. The remaining 72 subjects did not receive surgery for various reasons and thus could not be assigned risk; four other subjects undergoing surgery had no risk group assigned by the PI.

**TABLE 1:** Randomized subjects disposition including study metrics.

Subject disposition – all randomized subjects				
Number (%) of subjects	Group 1	Group 2	Group 3	Totals
Screened^a^	NA	NA	NA	1,236
Randomized	396	134	398	928
ITT exclusions^b^	1	0	4	5
ITT population n (%)	395 (100)	134 (100)	394 (100)	923 (100)
Safety population n (%)	383 (97.0)	129 (96.3)	367 (93.1)	879 (95.2)
Completed the study n (%)^c^	340 (86.1)	113 (84.3)	333 (84.5)	786 (85.2)
Primary reason for withdrawal n (%)				
Consent withdrawn	18 (4.6)	7 (5.2)	23 (5.8)	48 (5.2)
Lost to follow-up	25 (6.3)	7 (5.2)	20 (5.1)	52 (5.6)
Died n (%)^d^	204 (51.6)	68 (50.7)	190 (48.2)	462 (50.1)
Locoregional control failure n (%)	109 (27.6)	40 (29.9)	104 (26.4)	253 (27.4)
Progressed n (%)	230 (58.4)	78 (58.2)	216 (54.8)	524 (56.8)
Median follow-up (months)^e^	55.95	55.61	55.93	55.95
Time (days) since randomization to surgery				
N	361	123	367	851
Mean	36.0	35.3	13.0	26.0
Median	35.0	35.0	12.0	33.0
High risk evaluated post surgery n (%)	200 (50.6)	69 (51.5)	198 (50.3)	467 (50.6)
Low risk evaluated post surgery n (%)	158 (40.0)	54 (40.3)	168 (42.6)	380 (41.2)
Number of subjects				
Completed study-planned surgery n (%)	361 (91.4)	123 (91.8)	367 (93.1)	851 (92.2)
Completed study-planned RTx or CRTx) n (%)	328 (83.0)	115 (85.8)	342 (86.8)	785 (85.0)
Survived 36 months n (%)	208 (52.8)	78 (58.2)	228 (57.8)	514 (55.7)
Survived 60 months n (%)	177 (11.1)	63 (12.7)	191 (14.5)	431 (12.8)

Abbreviations: CRTx, chemoradiotherapy; ITT, intent-to-treat; RTx, radiotherapy.

Note: Percentages for randomized are calculated based on the number of randomized subjects. All subsequent are calculated based on ITT, population.

^a^ Total number of subjects screened includes non-randomized subjects.

^b^ Total ITT exclusions five [5] patients: four patients [4] warzone/hospital destruction could not be followed; one [1] signed/completed Case Report Form not available.

^c^ Completed the study refers to subjects that died at any time, irrespective of when the death occurred, and subjects that were alive at 35.5 months or longer.

^d^ From randomization date to the last follow up date or death date if died.

^e^ From randomization date to the last follow up date for those last alive.

Group 1 = LI + CIZ + SOC; Group 2 = LI + SOC; Group 3 = SOC only (Control).

The ITT median follow-up was 56 months for all three treatment groups; longest follow-up was 113 months. Most patients (85.2%) completed 36 months follow-up including deaths prior to month 36. A total of 462 deaths were reported; 461 subjects last reported alive–end of study, and 524 PFS events and 253 LRC failures were documented. There were 879 ITT subjects treated with the investigational study drug or SOC alone (Supplementary Figure S1), and 380 ITT LR subjects (41.2%) (Supplementary Figure S2) and 467 ITT HR subjects (50.6%) (Supplementary Figure S3) were identified at surgery.

### Characteristics of the ITT and ITT LR populations

Subject baseline demographics and characteristics (Supplementary Table S1) were comparable among randomization groups, ITT (n = 923) and ITT LR (n = 380). For the respective ITT and LR ITT populations, mean ages were 56.6/57.3 years, nearly 80% male, 79.7%/82.7% Caucasian (<2% black), percents from Europe/Eurasia 41.4%/51.3%, percents with tongue as primary were 45.8%/42.9%, percents AJCC Stage III 56.4%/68.9%, respectively. LR percent receiving RTx 92.6% (9 LR subjects received CRTx, physician’s choice); HR percent receiving CRTx was 91% (42 HR subjects received RTx, physician’s choice). Treatment regimens were well balanced (for ITT and ITT LR) with no significant differences between treatment groups.

There were minimal differences between treatment groups for tumor grade, nodal grade, and disease stage at screening for the ITT and ITT LR populations (Supplementary Table S2).

### Surgical findings

The percent with positive surgical margin was significantly lower for LR [0.26% (1/380)] vs. HR [30.4% (142/467)], and extracapsular nodal spread lower for LR [0.5% (2/380)] vs. HR [43.9% (205/467)]. These significant differences alone would preclude LR and HR pooling for analysis purposes (results were analyzed per ITT – see below).

### Overall survival results

**FIGURE 2:**
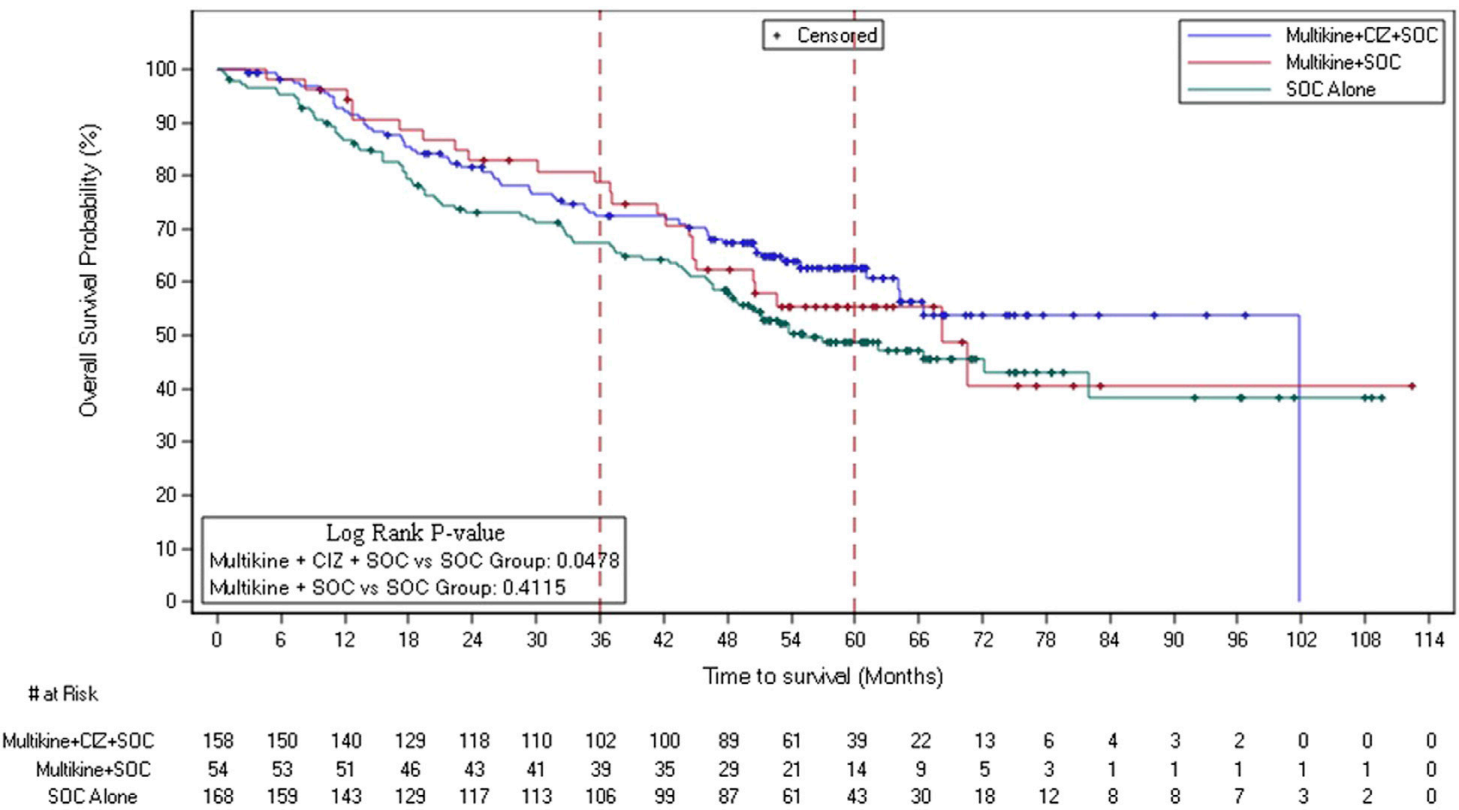
Overall survival Kaplan-Meier plot of ITT low-risk patients in the 3 treatment groups (n = 380). Abbreviations: CIZ, cyclophosphamide, indomethacin, zinc; ITT, intent-to-treat; OS, overall survival; SOC, standard of care. Multikine, LI (Leukocyte Interleukin, Injection). NOTE: blue line depicts OS for Group 1 (LI + CIZ + SOC), which at all times separated from and above Group 3 (SOC only, control) curve (green), red line depicts OS for Group 2 (LI + SOC).

Although statistical significance was not achieved for OS [n = 923, 462 deaths, two-sided unstratified log-rank (ULR) p = 0.41] in the overall ITT population, an OS advantage in favor of Group 1 (LI + CIZ + SOC) vs. Group 3 (SOC alone) in the ITT LR population was observed using Kaplan-Meier lifetables in the prospectively defined groups (two-sided ULR p = 0.048). The ITT LR Kaplan-Meier lifetable displays increasing separation over time between treatments (Figure 2). The absolute OS advantage for Group 1 vs. Group 3 in the ITT LR population was 4.9% (72.4%; 67.5%) at 3 years, 9.5% (76.3%; 57.8%) 4 years and 14.1% (62.7%; 48.6%) 5 years; the Cox proportional hazard ratio was 0.68 (95% CI: 0.48–0.95, Wald p = 0.024).

The ITT LR survival advantage was supported by the OS medians: Group 1 median OS 101.7 months, Group 2 68.2 months, and Group 3 (control) 55.2 months (a 46.5-month median OS advantage of Group 1 over Group 3). The ITT LR OS advantage was further confirmed in the ITT LR patients having stage III disease (ULR p = 0.047) and was favorable for the disease-directed therapy ITT LR RTx subset (ULR p = 0.079) (Supplementary Figures S4, S5 *, respectively,* Supplementary Material).

In contrast to the ITT LR population, in ITT HR patients there was a significant advantage for Group 3 vs. Group 1 (n = 467; 271 deaths), two-sided URL p = 0.046. The OS disadvantage of Group 1 in the HR population was likely due to the 3 weeks delay from randomization to surgery required for LI administration to LI subjects as opposed to HR controls whose surgery could occur sooner and the more severe disease in HR vs. the LR subjects [31–34]. Specifically, the hazard ratio associated with waiting an extra 3 weeks to surgery for the HR LI-treated regimens was 2.647 (two-sided Wald p = 0.040) in contrast to 1.109 for the overall ITT population with a significant 0.68 (two-sided Wald p = 0.023) hazard ratio for the LR (ITT) in the LI-treated regimen; indicating HR subjects with more severe disease could not afford to wait the extra 3 weeks to surgery.

### Pre-surgery objective early response and its impact on death rate, hazard ratio, and survival

RECIST v1.0 criteria were followed [35]. A total of 45 (PR/CR) OERs observed following the 3-week LI treatment regimen prior to surgery, were confirmed at surgery by pathology. Five CRs were reported among 45 responders, confirming tumor shrinkage seen in earlier LI published studies [22, 23]. No OERs were reported in the SOC alone group, consistent with no reported spontaneous tumor regressions in this population in the scientific literature.

The 45 OERs constituted 8.5% (45/529) of the ITT LI-treated subjects (Group 1 and Group 2) and was noted only in the LI-treated subjects (Table 2). The response rates were 16.0% (34/212) among ITT LR LI-treated, 3.7% (10/269) among ITT HR LI-treated, and 2.1% (1/48) among ITT LI-treated not classified as either LR or HR in the trial. Most early responders occurred among the ITT LR LI-treated subjects. Furthermore, significantly lower death rates and significantly lower hazard ratios were observed for responders (Table 3). Consistent >50% relative reductions in subsequent death rate were observed for LI responders.

**TABLE 2:** Objective early responders (n = 45) by treatment.

*Treatment arm (ITT, N = 923)*	Complete response before surgery (N = 5)		Partial response before surgery (N = 40)	
	*RECIST v1.0*	*Additional by pathology*	*RECIST v1.0*	*Additional by pathology*
Group 1: LI + CIZ + SOC (N = 395)	2	3	27	0
Group 2: LI + SOC (N = 134)	0	0	13	0
Group 3: SOC alone (N = 394)	0	0	0	0

Abbreviations: CIZ, cyclophosphamide, indomethacin, zinc; ITT, intent-to-treat; LI, leukocyte interleukin, Injection; SOC, standard of care.

**TABLE 3:** Pre-surgical response rates, death rates, and hazard rates in responders vs. non-responders.

Treatment group	Site determined risk classification			
	Early response rate			
	Low risk	High risk	Unclassified	Totals
LI + CIZ + SOC	24/158 (15.2%)	7/200 (3.5%)	1/37 (2.7%)	32/395 (8.1%)
LI + SOC	10/54 (18.5%)	3/69 (4.3%)	0/11 (0%)	13/134 (9.7%)
Combined LI	**34/212 (16.0%)**	**10/269 (3.7%)**	**1/48 (2.1%)**	**45/529 (8.5%)**
SOC alone (control)	**0/168 (0%)**	**0/198 (0%)**	**0/28 (0%)**	**0/394 (0%)**
2-sided Fisher’s exact test	p < 0.0001	p = 0.0062	NS	p < 0.0001

	Death rate			
	Combined LI	LR combined LI	LR LI + CIZ + SOC	
Response outcome				
Responders	10/45 (22.2%)	6/34 (17.6%)	3/24 (12.5%)	
Non-responders	262/484 (54.1%)	76/178 (42.7%)	55/134 (41.0%)	
2-sided Fisher’s exact test	p < 0.0001	p = 0.0068	p = 0.01	

	Hazard ratio			
	Combined LI	LR combined LI	LR LI + CIZ + SOC	
Hazard ratio	0.301	0.348	0.246	
2-sided 95% CI	0.16–0.566	0.152–0.801	0.077–0.787	
2-sided Wald test	p < 0.0001	p = 0.0067	p = 0.01	

Abbreviations: CIZ, cyclophosphamide, indomethacin, zinc; LI, leukocyte interleukin, Injection; LR, low risk; NS, not-significant; SOC, standard of care. Bold values are the results used for the analysis of statistical significance presented in the table.

**FIGURE 3:**
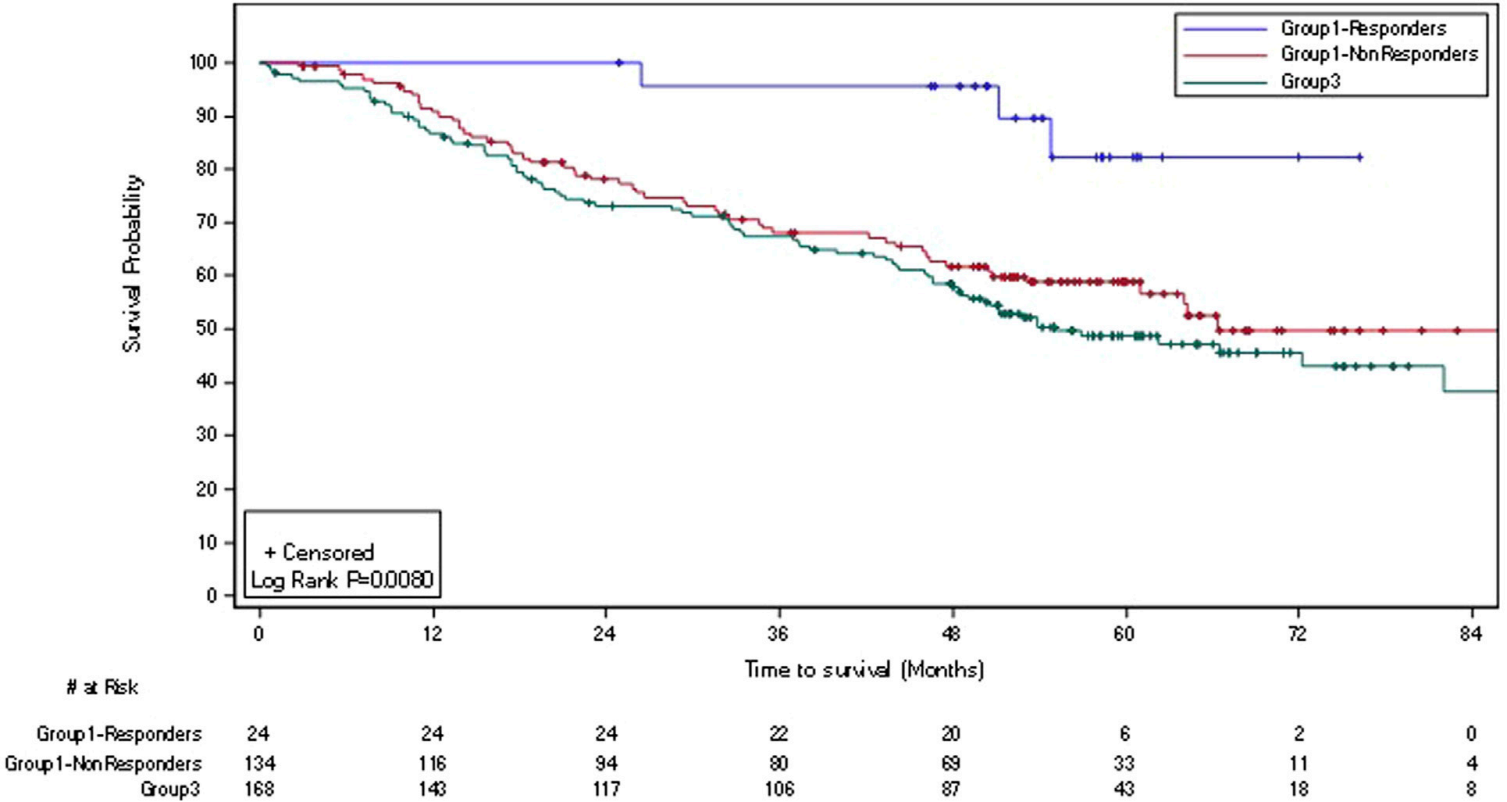
Kaplan-Meier plot of overall survival in ITT low-risk responding* and non-responding subjects treated with LI + CIZ + SOC and SOC alone (n = 326). Abbreviations: CIZ, cyclophosphamide, indomethacin, and zinc as multivitamin supplement; ITT, intent-to-treat; LI, Leukocyte Interleukin, Injection; SOC, standard of care. *Response per RECIST v.1.0 (and confirmed by pathology at surgery).

OS was significantly more favorable for LR Group 1 responders vs. LR Group 1 non-responders (2-sided ULR p = 0.010) and vs. LR Group 3 (2-sided ULR p = 0.002) (Figure 3); LR Group 1 non-responders had more favorable OS vs. LR Group 3 suggesting carryover benefit beyond response. ITT LR responders experienced >30% absolute higher survival at months 36, 48, and 60 than non-early responders as well as vs. SOC. Overall, ITT and LR ITT responders experienced >25% absolute higher survival at months 36, 48, and 60 vs. both non-responders and SOC (Table 4).

**TABLE 4:** OS of ITT Group 1 OERs, ITT Group 1 non-OERs, and ITT Group 3 (N = 789) [upper panel] and OS of ITT LR Group 1 OERs, LR Group 1 non-OERs, and LR Group 3 (N = 326) [lower panel].

Time to survival (months)	ITT Group 1 (N = 395)			Comparisons	
	(A) OERs (N = 32)	(B) Non-OERs (N = 363)	(C) ITT Group 3 (N = 394)		
	OS% [95% CI]	OS% [95% CI]	OS% [95% CI]	(A) vs. (C)	(B) vs. (C)
12	100.0 [89.5–100.0]	80.4 [75.7–84.2]	81.9 [77.5–85.5]	+18.1	−1.5
24	90.0 [72.1–96.7]	64.3 [58.9–69.1]	67.5 [62.4–72.1]	+22.5	−3.3
36	86.5 [68.0–94.7]	53.5 [47.9–58.7]	60.6 [55.2–65.5]	+26.0	−7.1
48	83.1 [64.0–92.6]	45.9 [40.3–51.2]	53.7 [48.3–58.8]	+29.4	−7.9
60	72.9 [50.3–86.4]	41.0 [35.4–46.5]	47.7 [42.2–53.0]	+25.2	−6.7
Median OS (months)	NR [NR–NR]	42.1 [34.6–50.8]	52.9 [46.5–66.6]	NR	−10.8
Hazard ratio [95% CI]				0.332 [0.156–0.709]	1.182 [0.968–1.445]

Time to survival (months)	LR Group 1 (N = 158)			Comparisons	
	(A) OERs (N = 24)	(B) Non-OERs (N = 134)	(C) LR Group 3 (N = 168)		
	OS% [95% CI]	OS% [95% CI]	OS% [95% CI]	(A) vs. (C)	(B) vs. (C)
12	100.0 [85.8–100.0]	90.7 [84.2–94.6]	86.8 [80.6–91.1]	+13.2	+3.9
24	100.0 [85.8–100.0]	78.0 [69.8–84.3]	73.2 [65.7–79.3]	+26.8	+4.8
36	95.7 [72.9–99.4]	68.0 [59.0–75.4]	67.5 [59.7–74.1]	+28.2	+0.5
48	95.7 [72.9–99.4]	61.8 [52.6–69.8]	57.8 [49.7–65.0]	+37.9	+4.0
60	82.2 [53.1–94.1]	58.8 [49.3–67.0]	48.6 [40.4–56.4]	+33.6	+10.1
Median OS (months)	NR [NR–NR]	66.4 [53.1–101.7]	55.2 [48.0–NR]	NR	+11.2
Hazard ratio [95% CI]				0.169 [0.053–0.539]	0.785 [0.555–1.110]

Abbreviations: CIZ, cyclophosphamide, indomethacin, zinc; ITT, intent-to-treat; LI, leukocyte interleukin, Injection; LR, low risk; NR, not reached; OER, objective early response; OS, overall survival; SOC, standard of care.

Group 1 = LI + CIZ + SOC; Group 3 = SOC (control).

Table 5 summarizes the efficacy of LI + CIZ + SOC vs. SOC in ITT low-risk cases for each of OS, PFS, and LRC; the OS advantage (0.68 hazard ratio) extends to PFS (0.76 hazard ratio) and LRC (0.84 hazard ratio) in the ITT low-risk population. Importantly, the median OS for LI + CIZ + SOC, LI and SOC was 101.7, 68.2, and 55.2 months, respectively, demonstrating the need for CIZ as a modulator of immune activation by LI pointing to the superior efficacy of neoadjuvant LI + CIZ + SOC, in the locally advanced treatment-naïve, resectable OSCC (oral cavity and soft palate) patients, over SOC alone.

**TABLE 5:** ITT low-risk summary: study entry to exit.

	Treatment comparison	OS (380; 166d)	PFS (380; 188p)	LRC (380; 104f)
	Failures (Group “1,” “2,” “3”)^a^	(58, 24, 84)	(70, 27, 91)	(41, 16, 47)
ULR p-value	LI + CIZ + SOC vs. SOC	0.0478	0.1797	0.6142
SLR p-value	LI + CIZ + SOC vs. SOC	0.0137	0.0159	0.3024
Hazard ratio	LI + CIZ + SOC vs. SOC	0.68 (0.48–0.95)	0.76 (0.54–1.04)	0.84 (0.55–1.28)
	LI + SOC vs. SOC	0.82 (0.52–1.29)	0.84 (0.54–1.30)	0.93 (0.53–1.65)
Cox PH p-value	LI + CIZ + SOC vs. SOC	0.0236	0.0896	0.4082
	LI + SOC vs. SOC	0.3859	0.4376	0.8131
Median (months)	LI + CIZ + SOC	101.7 months	66.4 months	Not reached
	LI + SOC	68.2 months	68.2 months	Not reached
	SOC	55.2 months	51.2 months	Not reached

Cox model included treatment (SOC referent), tumor stage, tumor location, and geographic location.

Abbreviations: d, deaths; p, progressions; f, failures; LRC, locoregional control; OS, overall survival; PFS, progression-free survival; SLR, stratified log-rank; ULR, unstratified log-rank.

^a^ Group “1” = LI + CIZ + SOC; Group “2” = LI + SOC; Group “3” = SOC, alone.

### AJCC stage changes and survival correlates

The screening/entry AJCC stage distribution and its changes during treatment were also investigated in relation to risk groups, response and survival. There was a significant difference in the AJCC stage distribution between LR and HR at screening/entry (two-sided generalized Fisher’s exact test p < 0.0001); the AJCC stage was more favorable (i.e., showed less disease burden) at screening/entry for subjects subsequently deemed (post-surgery) as LR (per NCCN guidelines) (Supplementary Table S3).

The AJCC stage distributions were compared at screening/entry (baseline) for responders vs. non-responders within each LI treatment to rule out differences or trends favoring AJCC stage baseline for responders vs. non-responders for each study group. Overall treatment group differences were observed for the AJCC stage considered as being “worse,” “same,” or “improved” (“better”) (from screening to surgery), but there was an advantage in the “improved” AJCC (TN) stage favoring the LI treatments vs. SOC (Table 6). In the overall ITT population, Group 1 (LI + CIZ + SOC) saw an absolute 5.2% increase in pre-surgery downstaging (PSD) vs. Group 3 (SOC control) (25.6% vs. 20.4%). As OER, the PSD rate increase was greater in the ITT LR Group 1 with 12.0% more PSD vs. control (38.9% vs. 26.9%, p = 0.0210).

**TABLE 6:** AJCC stage change distributions from screening to surgery.

	Group 1 (LI + CIZ + SOC)	Group 2 (LI + SOC)	Group 3 (SOC)	Group 1 (%) vs. Group 3 (%)
Overall ITT study population				
Number of subjects w/TN scores, N	347	115	353	
Downstage % (n)	**25.6 (89)**	**27.0 (31)**	**20.4 (72)**	**+5.2%**
No change % (n)	42.7 (148)	40.9 (47)	49.6 (175)	−6.9%
Upstage % (n)	31.7 (110)	32.2 (37)	30.0 (106)	+1.7%
*p*-value vs. Group 3^a^	0.5653	0.6139	N/A	
Low-risk cohort				
Number of subjects w/TN scores, N	144	49	160	
Downstage % (n)	**38.9 (56)**	**44.9 (22)**	**26.9 (43)**	**+12.0%**
No change % (n)	51.4 (74)	46.9 (23)	58.8 (94)	−7.4%
Upstage % (n)	9.7 (14)	8.2 (4)	14.4 (23)	−4.7%
p-value vs. Group 3^a^	0.0210	0.0176	N/A	
High-risk cohort				
Number of subjects w/TN scores, N	199	66	192	
Downstage % (n)	**16.1 (32)**	**13.6 (9)**	**15.1 (29)**	**+1.0%**
No change % (n)	37.2 (74)	36.4 (24)	41.7 (80)	−4.5%
Upstage % (n)	46.7 (93)	50.0 (33)	43.2 (83)	+3.5%
p-value vs. Group 3^a^	0.6586	0.3977	N/A	

^a^ Two-sided Fisher’s exact test of PSD/no-PSD, Group 1 vs. Group 3; PSD, rates are bolded for comparison.

Abbreviations: ITT, intention to treat; TN, tumor-lymph node; PSD, pre-surgery downstaging.

Bold values are the results used for the analysis of statistical significance presented in the table.

**FIGURE 4:**
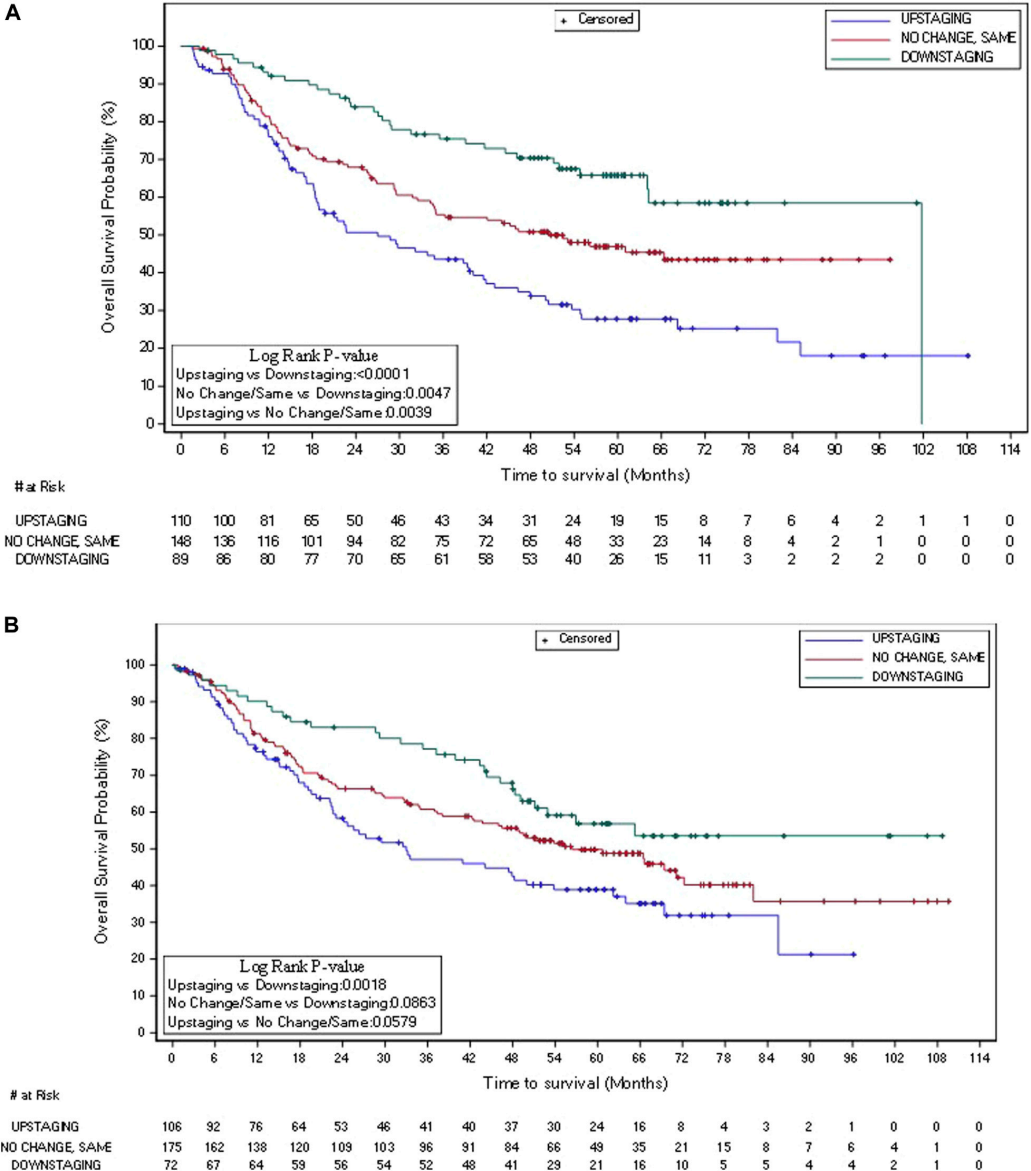
Kaplan-Meier plot of overall survival in overall ITT Group 1 **(A)** (N = 347) and Group 3 **(B)** (N = 353): AJCC (TN) PSD, no change, and upstage.

Literature supports PSD (per AJCC staging) after neoadjuvant therapy as a surrogate for OS in SCCHN [26]. In this study, AJCC downstaging had significant OS advantage vs. both no change and upstaging. In Group 1 (n = 347), PSD (AJCC downstaging) vs. upstaging hazard ratio was 0.405 (95% CI: 0.272–0.602, Wald p < 0.0001). Kaplan-Meier OS curves for PSD vs. no stage change (ULR p = 0.0047) and vs. upstaging for ITT Group 1 (ULR p < 0.0001) are shown in Figure 4A. Downstaging resulted in consistent survival benefit for Group 1 [>35% absolute (38%) survival advantage, at 5 years], with a <20% absolute advantage [18%] at 5 years in Group 3. However, the differences in Group 3 were lower than in Group 1 between downstaging, no change and upstaging for OS (Figure 4B). Overall ITT OS for Group 1 PSDs at 3 and 5 years was >75% and >65%, respectively (Figure 4B).

### Other efficacy endpoints

While there were no significant LI + CIZ + SOC advantages over SOC alone for PFS and LRC in the overall population, a favorable trend was observed for the ITT LR group in the case of the PFS (Supplementary Figure S6), with a hazard ratio of 0.76 (95% CI: 0.55–1.04). The LRC did not show difference in LR cases either; hazard ratio was 0.84 (95% CI: 0.55–1.28). For LR PFS, consistent separation was noted as early as month 12 in support of efficacy (Supplementary Figure S7).

### Quality of life measures

The QoL (Supplementary Table S4) improved the least for the non-responders (83.4% best two scores), more for the PR/CRs (89.4% best two scores), and most for the CRs (95.1%); the CRs had 39 (60%) of a total of QoL 65 measures rated at 100% (best ordinal score). The CR data was based on an average of 11.7 post-baseline evaluations which corresponds to >3 years of follow-up. Among the five CRs, 95% of all responses were classified as the “good outcome” showing sustained QoL benefit.

### Safety

The ITT safety population consisted of a total of 94.7% (879/923) treated subjects who received at least one study directed treatment; this excludes 44 untreated ITT subjects without surgery. Of the 529 ITT subjects randomized to LI treatment, 95.3% (504/529) received at least one dose of LI. Among the maximum possible LI injections (15,870 injections to all subjects randomized to LI), 92.7% were administered; 90.0% receiving all planned dosing, and 94.7% (485/529) receiving >90% of planned doses. Twenty-five LI subjects (20 Group 1; 5 Group 2) were randomized to LI treatment but never received LI for various reasons. Safety was assessed by intervals from signed informed consent to pre-surgery (LI administration period); during DDT (i.e., during SOC), post-SOC and from study entry to exit.

Pre-surgery TEAEs were all expected events. Excluding deaths, recurrences, and progressions, the most frequently reported events were as follows:

• Group 1 TEAEs:
  ◦ injection site hemorrhage [5.2% (20/383); 40 events]
  ◦ injection site pain [6.0% (23/383); 27 events]
• Group 2 TEAEs:
  ◦ oral pain [6.2% (8/129); 8 events]
  ◦ injection site hemorrhage [6.2% (8/129); 12 events], and
  ◦ injection site pain [6.2% (8/129); 12 events].

All of these AEs were expected, all resolved, and none resulted in the delay or completion of surgery or subsequent DDT (Supplementary Table S5). No pre-surgery TEAEs persisted following surgery. Results for the LR group were consistent with those in the HR group and the overall safety population. No TEAEs (other than progression or recurrence) in LI-treated subjects led to death (Supplementary Table S6).

Other than the post-entry/pre-surgery interval where more TEAEs were observed for Groups 1 and 2 than Group 3 (of note: Group 3 did not receive any treatment between entry and surgery), there was near parity in TEAE incidence post-surgery. Post-entry/pre-surgery (Supplementary Figure S8) refers to events with a start date prior to surgery.

## Discussion

This randomized controlled study was designed to confirm the previously observed Phase 2 safety and efficacy of LI in treatment naïve resectable subjects with locally advanced primary SCCHN oral and soft palate squamous cell carcinoma.

There were no safety signals or issues noted in this or previous studies with LI. Approximately 66% of subjects with SCCHN present with locally advanced disease [36, 37] at their first visit (diagnosis), and expect poor prognosis, OS <50% at 5 years.

Our meta-analysis based on 66 studies (2009–2021) of LA primary SCCHN subjects showed that the 3-year OS for locally advanced subjects (all head and neck tumor locations) was 64.57% (7 studies; 1,258 cases; 95% CI: 55.52%–73.61%) and the 5-year OS was 46.61% (9 studies; 6,209 cases; 95% CI: 42.04%–51.17%). In the IT-MATTERS study reported here the control group had more favorable survival (48.7% at 5 years).

Furthermore, we reviewed the Surveillance, Epidemiology, and End Results (SEER) Database 18 for 2000 to 2016 (n = 6,569) where data from 2000 to 2010 (n = 3,291) were excluded, to “match” the IT-MATTERS timing of accrual/treatment interval (2011–2016) (used SEER Stat Section 8.3.5). A set of subjects in SEER Database 18 matched the IT-MATTERS study subjects’ disease stage and tumor location at entry. The SEER OS for the combined stage III and IVa subjects (n = 3,278) was 46.6% at 3 years, and 36.8% at 5 years. The SEER subjects were treated with the same SOC as in the IT-MATTERS study. Thus, we conclude that the 48.7% OS at 5-year ITT LR SOC in this study outperformed both the meta-analysis and SEER data for a comparable patient population at 5 years supporting study credibility and further underscoring the superiority of LI in LR cases vs. contemporaneous randomized LR control.

The study results are the first demonstration in a randomized controlled pivotal study of the utility of short-term locally administered neoadjuvant immunotherapy to achieve OERs, AJCC classification downstaging, and extending OS in the ITT LR group. Despite OS improvement in other cancers, no increase in OS has been achieved by the administration of the current SOC for LR for patients with recurrence in decades, underscoring the unmet need of this patient population.

Objective early (pre-surgery) response was particularly meaningful because it was achieved with a 3-week LI therapy cycle and responses were confirmed by pathology at surgery; no subjects died before surgery, so the OS results include all subjects. There are no known/reported spontaneous regressions in treatment naïve resectable locally advanced primary squamous cell carcinoma of the oral cavity/soft palate. Unlike other oncology studies where response occurs after initiation of DDT, LI neoadjuvant treatment leads to an isolated (from other treatments) pre-surgical response. The 45 responders included five CRs all achieved within 3–5 weeks, with minimal toxicity relative to neoadjuvant chemotherapies for all other solid tumors.

The objective early (pre-surgery) response impacted the ITT LR population (n = 380). This pre-defined group had 81% power to detect the 14.1% OS advantage at 5 years (62.4% vs. 48.7%). The OS advantage had a 0.68 hazard ratio (Wald two-sided p = 0.026) and a median OS advantage of 46.5 months [101.7 (ITT LR LI) vs. 55.2 (ITT LR SOC control)]. The 0.68 hazard ratio equates to a 47% OS prolongation. Responders were confirmed to have pre-surgical downstaging (AJCC migration) shift from entry to surgery, with ITT Group 1 having a small (5.2%) increase in PSD vs. ITT control (25.6% vs. 20.4%). As with objective early (pre-surgery) response, the PSD rate increase was greater in the ITT low-risk Group 1, having 12.0% more PSD vs. control (38.9% vs. 26.9%, p = 0.0210). Furthermore, our results demonstrate that baseline AJCC staging could provide markers for the selection of OSCC/soft palate patients for LI treatment in future studies, which could be used to distinguish between LR and HR patients at screening/entry. Likewise, the downstaging (shift from entry to surgery) of the AJCC stage, in response to LI pre-surgery treatment, resulted in improved OS vs. upstaging [unstratified log-rank (ULR) p < 0.0001] and vs. no change (ULR p < 0.0047). Therefore, we suggest that patients with reduced disease burden prospectively identified at entry by PET-CT/MRI imaging could benefit from neoadjuvant LI-treatment.

The ITT high-risk patients were shown to have significantly more severe tumor and nodal involvement than the ITT low-risk patients at screening/entry, which points toward using current imaging methods (including PET-CT/MRI imaging) to identify the adverse features characterizing high risk (as currently are determined at surgery). Therefore, in contrast to the low-risk group, the high-risk group was not able to benefit from neoadjuvant LI treatment since the treatment requires 3 consecutive weeks of daily (5x/week) delivery. This resulted in a median 3 additional weeks delay (a total of 5 weeks or 35 days) to surgery for the high-risk LI-treated patients compared to median time to surgery (11 days) for high-risk SOC. The impact of delay in the time to treatment initiation in SCCHN in general is an independent predictor of decreased OS, and has a negative impact on locally advanced OSCC, where even a 20-day delay to surgery in oral cavity SCC has been shown to have a detrimental impact on OS [31, 34, 38]. High-risk subjects treated with LI had thus a decreased OS vs. high-risk controls whose median time to surgery was 24 days shorter than that of high-risk LI-treated patients. The decreased OS is thought to be due to the surgery delay.

Validated QoL instruments, EORTC QLQ-C30 and EORTC QLQ-H&N 35, were used in this study. These same instruments are used by the Radiation Therapy Oncology Group [39, 40], per the NCCN Guidelines for advanced SCCHN. The QoL for CRs had an average of 11.7 post-baseline evaluations corresponding to >3 years follow-up. The most notable improvements included resolution of eating/swallowing problems, mouth pain/soreness, sense of taste, food rejection, weakness, trouble sleeping, and social interaction, all of which achieved 100% “good outcome” rating post-baseline and following neoadjuvant LI treatment through to an average exceeding 3-year post treatment.

The safety of LI was confirmed; LI treatment demonstrated >85% reduction in AEs vs. subsequent DDT. The percent with TEAEs post-surgery was comparable among all treated groups. No safety issues were reported for LI treatment in addition to those derived from the SOC treatment vs. SOC alone. LI treatment did not interfere with administration of surgery or subsequent DDT. Neoadjuvant LI treatment resulted in improved outcomes, without addition of treatment burden, while greatly improving and sustaining the QoL for LI-treated responders in a population for which no improved outcomes have been forthcoming in decades. In addition, LI neoadjuvant treatment resulted in objective early responses (OERs) [complete responses (CRs)/partial responses (PRs)] per RECIST v1.0, confirmed by pathology, significantly reduced death rate and increased OS in LI-responders vs. non-responders and as compared to OS and death rate in control (SOC alone).

LI is the first neoadjuvant immunotherapy to achieve objective early responses (CRs/PRs – confirmed by pathology) and show AJCC downstage shift from randomization to surgery leading to improved OS outcome in the resectable, treatment-naïve locally advanced primary OSCC and soft palate SCC without safety issues or adding treatment burden over SOC alone. The IT-MATTERS study represents the first advance in OS in the treatment of these patients in many decades.

## Data Availability

The original contributions presented in the study are included in the article/Supplementary Material, further inquiries can be directed to the corresponding author.

## Group members of the IT-MATTERS Study Working Group

Mikhail Dvorkin, Omsk Regional Oncology Dispensary, Omsk, Russian Federation; Dmitry Bentsion, Sverdlovsk Regional Oncology Center, Ekaterinburg, Russian Federation; Vladyslav Bakhtalovskiy, Communal Institution of Healthcare “Kharkiv Regional Clinical Oncological Center,” Department of Head and Neck Tumour, Kharkiv, Ukraine; Milovan Dimitrijevic, Clinical Centre Serbia, Clinic for ORL and Maxillofacial Surgery, Belgrade, Serbia; Viktor Paramonov, Communal Institution “Cherkasky Regional Oncological Dyspensary” Cherkasy Regional Board, Department of Head and Neck Tumour, Cherkasy, Ukraine; Aleksandar Kiralj, Clinical Centre Vojvodina, Clinic for Maxillofacial Surgery, Novi Sad, Serbia; Jayamini Horadugoda, Karapitiya Teaching Hospital, Galle, Sri Lanka; Dragan Krasic, Clinic for Stomatology Nis, Department for Maxillofacial Surgery, Nis, Serbia; Yaroslav Shparyk, Lviv State Oncology Regional Treatment and Diagnostic Centre, Lviv, Ukraine; Evgeniya Karchmit, Healthcare Institution “Vitebsk Regional Clinical Oncology Dispensary,” Vitebsk, Belarus; Rajnish Vasant Nagarkar, Department of Surgical Oncology, HCG Manavata Cancer Centre, Nashik, India; Olena Sukhina, State Institution “Medical Radiology and Oncology Institute named after S. P. Hryhoriev of the National Academy of Medical Sciences of Ukraine,” Department of Remote, Combined Radiation and Complex Therapy, Kharkiv, Ukraine; Prasad Abeysinghe, National Cancer Institute, Maharagama, Sri Lanka; Dinko Leovic, Clinical Hospital Center Osijek, Osijek, Croatia; Ravi Rejnish Kumar, Regional Cancer Centre, Department of Radiation Oncology, Trivandrum, India; Devendra Chaukar, Tata Memorial Hospital, Mumbai, India; Jovica Milovanovic, Clinical Centre Serbia, Clinic for ORL and Maxillofacial Surgery, Belgrade, Serbia; Tarik Masic, Clinical Center University of Sarajevo, Clinic for Maxillofacial Surgery, Sarajevo, Bosnia and Herzegovina; Suresh Attili, Bibi General Hospital and Cancer Centre, Hyderabad, India; Sławomir Okła, Świętokrzyskie Centrum Onkologii, Dział Otolaryngologii, Chirurgii Głowy i Szyi, Kielce, Poland; Jacek Fijuth, Wojewódzkie Wielospecjalistyczne Centrum Onkologii i Traumatologii im. M. Kopernika, Zakład Teleradioterapii, Łódź, Poland; Ku-Hao Fang, Chang Gung Medical Foundation – Linkou Branch Chang Gung Memorial Hospital, Taoyuan County, Taiwan; Subramania Iyer, Amrita Institute of Medical Sciencies and Research Centre, Kochi, India; Nagarajan Murugaiyan, G. Kuppuswamy Naidu Memorial Hospital, Coimbatore, India; Sanja Spiric, University Clinical Centre of the Republic of Srpska, Clinic for ENT and Maxillofacial Surgery, Banja Luka, Bosnia and Herzegovina; Éva Remenár, National Institute of Oncology, Multidisciplinary Head and Neck Cancer Center, Budapest, Hungary; Janusz Klatka, Klinika Otolaryngologii i Onkologii Laryngologicznej, SPSK nr 4 w Lublinie, Lublin, Poland; Misko Zivic, MD, Clinical Hospital Nis, Clinic for ORL, Nis, Serbia; Kosanam Subramanian Kirushna Kumar, Meenakshi Mission Hospital & Research Centre, Madurai, India; Balaji Shewalkar, Marathwada Regional Cancer Centre and Research Insititute, Aurangabad, India; Raphel Feinmesser, Tel Aviv University, Rabin Medical Center, Petah Tikva, Israel; Stanislav Zolotukhin, Communal Clinical Institution “Donetsk Anticancer Center,” surgical department #5, Donetsk, Ukraine; Miro Leventic, University Clinical Hospital Mostar, Clinic for ENT and Maxillofacial Surgery, Clinic for Radiotherapy and Oncology, Mostar, Bosnia and Herzegovina; Mario Bilic, Clinical Hospital Center Zagreb, Zagreb, Croatia; Davorin Djanic, General Hospital Josip Bencevic, Slavonski Brod, Croatia; Zsolt Németh, Semmelweis University, Department Oral and Maxillofacial Surgery and Stomatology, Budapest, Hungary; József Piffkó, University of Szeged, Department of Oral and Maxillofacial Surgery, Szeged, Hungary; Rajendra Singh Arora, Sujan Surgical Cancer Hospital, Amravati, India; Sen-Tien Tsai, National Cheng Kung University Hospital, Tainan, Taiwan; Mu-Kuan Chen, Changhua Christian Hospital, Changhua County, Taiwan; Ming-Hsui Tsai, China Medical University Hospital, Taichung, Taiwan; Tibor Csőszi, Jász-Nagykun-Szolnok County Hospital, Szolnok, Hungary; Sandeep Agarwal, Galaxy Cancer Center, Ghaziabad, India; Rachmiel Adi, Rambam Healthcare Campus; Oral & Maxillofacial Surgery, Haifa, Israel; Mohd Razif Mohamad Yunus, University Kebangsaan Medical Centre, Kuala Lumpur, Malaysia; Alfredo Quintin Y. Pontejos, Philippine General Hospital, Manila, Philippines; Marcin Hetnał, MD, Rydygier’s Hospital, Radiotherapy Department, Kraków, Poland; Igor Lifirenko, Kursk Regional Clinical Oncological Dispensary, Kursk, Russian Federation; Pei-Jen Lou, National Taiwan University Hospital, Taipei, Taiwan; Patravoot Vatanasapt, Khon Kaen University, Khon Kaen, Thailand; Olga Ponomareva, Kyiv City Clinical Oncology Center of the Main Healthcare Department of Kyiv, Day Care Facility with beds for oncology patients, Kyiv, Ukraine; Vedran Uglesic, Clinical Hospital Dubrava, Zagreb, Croatia; Gilles Dolivet, Institut de Cancerologie de Lorraine, Vandoeuvre-les-Nancy Cedex, France; Baharudin Abdullah, Hospital Universiti Sains Malaysia, Kelantan, Malaysia; Mohd Zulkiflee Abu Bakar, University of Malaya Medical Centre, Kuala Lumpur, Malaysia; Philip Rajan, Hospital Raja Permaisuri Bainun, Ipoh, Malaysia; Andrzej Kawecki, Centrum Onkologii – Instytut im. Marii Skłodowskiej-Curie, Klinika Nowotworów Głowy i Szyi, Warszaw, Poland; Cornelia Toganel, Spital Clinic Judetean Mures, Targu Mures, Romania; Diana Petroiu, Regional Institute of Oncology, Iasi, Romania; Eugeny Kulikov, Ryazan' Regional Clinical Oncology Dispensary, Head and Neck Department, Ryazan, Russian Federation; Chen-Chi Wang, Taichung Veterans General Hospital, Taichung City, Taiwan; Nilda Suslu, Hacettepe University Faculty of Medicine, Ankara, Turkey; Ihor Vynnychenko, Sumy Regional Clinical Oncological Dyspensary, Oncothoracal Department, Sumy, Ukraine; Julian Hill, Hematology & Oncology Associates, Tupelo, United States; Samel Ejadi, UC Irvine Chao Family Comprehensive Cancer Center, Orange, CA, United States; Christopher Oliver, University of Colorado, Department of Otolaryngology, Aurora, CO, United States.

## Abbreviations

AE, adverse event; CIZ, cyclophosphamide, indomethacin, zinc; CR, complete response; CRT, chemoradiotherapy; CTLL, cytotoxic T lymphocyte (cell) line; DDT, disease directed therapy; FFPE, formalin-fixed paraffin-embedded; HR, high risk; ICI, immune checkpoint inhibitor; ITT, intent to treat; LA, locally advanced; LI, leukocyte interleukin; LR, low risk; LRC, locoregional control; NSCLC, non-small cell lung cancer; OER, objective early response; OS, overall survival; OSCC, oral squamous cell cancer; PBMCs, peripheral blood mononuclear cells; PFS, progression-free survival; PR, partial response; PSD, pre-surgery downstaging; QoL, quality of life; RT, radiotherapy; SCCHN, squamous cell cancer of head and neck; SOC, standard of care; TEAE, treatment emergent adverse event; TN, tumor-lymph node; ULR, unstratified log-rank.
