# Supplementary material for: Neoadjuvant leukocyte interleukin injection immunotherapy improves overall survival in low-risk locally advanced head and neck squamous cell carcinoma –the IT-MATTERS study
Source: Pathol Oncol Res. 2025 Mar 21;31:1612084. doi: 10.3389/pore.2025.1612084 (PMC11968324; doi:10.3389/pore.2025.1612084)
Supplement: Supplementary file 1 [file DataSheet2.pdf]

CHECKLIST FOR POST-OPERATIVE REPORT FOR NECK DISSECTION

Patient ID: [ ] **Patient Initials:** [ ]

Case Report Sheet[1] Number: [ ]

Clinical Notifications (CN1-9)

Clinical Setting (CN1-6)

NOTE for CN 1-4: Select All that Apply.

CN1: Clinical History

☐ Neoadjuvant Therapy

- ☐ Yes (specify type):

  For Multikine treatment specify area (lymph node level) of drug instillation:

  - ☐ Level I (submental [IA], submandibular [IB]
  - ☐ Level II (deep to ***upper*** third of the sternocleidomastoid muscle)
  - ☐ Level III (deep to the ***middle*** third of the sternocleidomastoid muscle)
  - ☐ Level IV (deep to ***lower*** third of the sternocleidomastoid muscle)
  - ☐ Level V (dorsal to the posterior border of the sternocleidomastoid muscle)
  - ☐ Level VI (midline area of the neck extending from the hyoid bone to the suprasternal notch)
  - ☐ Level VII (upper mediastinal lymph nodes)

☐ No

☐ Indeterminate

☐ Other (specify):

[1]A compilation by Glasz, T MD; 2nd Dept. of Pathology, Semmelweis University (Budapest, Hungary), based on relevant guidelines of the College of American Pathologists, The Royal College of Pathologists, AJCC/UICC TNM – 7th edition, as well as parts from Rosai and Ackerman's Surgical Pathology.

CHECKLIST FOR POST-OPERATIVE REPORT FOR NECK DISSECTION

CN2: Methods Performed for Metastatic Tumor Detection

☐ Visual Inspection

☐ Palpation

☐ Ultrasound

☐ Tomography (CT, MRI)

☐ Other (specify):

CN3: Metastatic Tumor Detection

☐ Evident on Visual Inspection

☐ Detectable on Palpation

☐ Detectable with Ultrasound

☐ Detectable with Tomography (CT, MRI)

☐ Metastatic disease is not evident with clinical detection methods

☐ Other (specify):

CN4: Metastatic Tumor Laterality

☐ Right

☐ Left

☐ Midline

☐ Not specified

☐ Uncertain

CHECKLIST FOR POST-OPERATIVE REPORT FOR NECK DISSECTION

NOTE for CN5: Select one (1)

CN5: Metastatic Tumor Focality

☐ Single focus

☐ Multifocal

NOTE for CN6: Select all that apply

CN6: Metastatic Tumor Site

☐ Level I (submental [IA], submandibular [IB]

☐ Level II (deep to the ***upper*** third of the sternocleidomastoid muscle)

☐ Level III (deep to the ***middle*** third of the sternocleidomastoid muscle)

☐ Level IV (deep to the ***lower*** third of the sternocleidomastoid muscle

☐ Level V (dorsal to the posterior border of the sternocleidomastoid muscle)

☐ Level VI (midline area of the neck extending from the hyoid bone to the suprasternal notch)

☐ Level VII (upper mediastinal lymph nodes)

☐ Soft tissue of the neck (specify):

☐ Vessel involvement (specify):

☐ Skin involvement

☐ Other (specify):

☐ None detected

☐ Not specified

CHECKLIST FOR POST-OPERATIVE REPORT FOR NECK DISSECTION

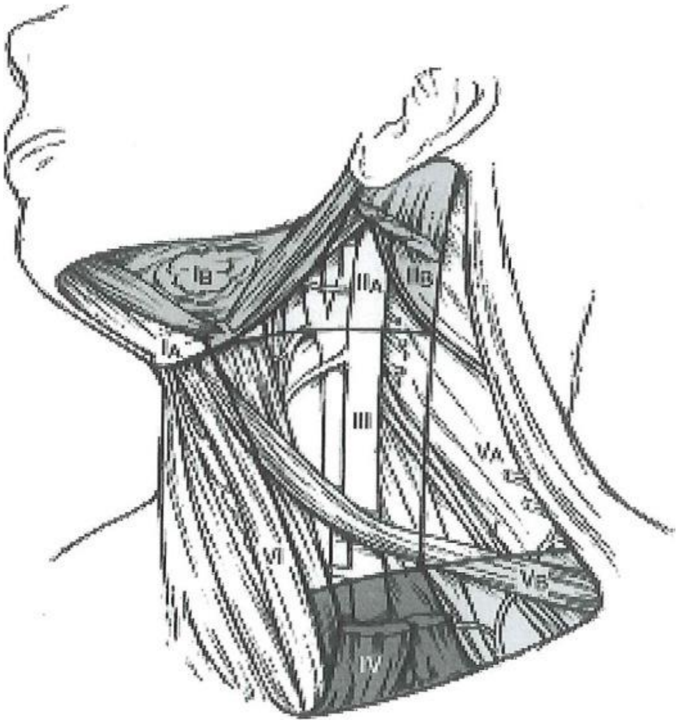

Tumor Sampling (CN7-9)

<u>NOTE:</u> For sections CN7-9, select all that apply. Identify with numbers if more than one specimen is taken. Any issue may receive more than one identification number. If numbers are given, they must be consistent here and in all subsequent sections of this Case Report Sheet with those defined in CN7.

CN7: Specimen Laterality

☐ Right

☐ Left

☐ Midline

☐ Not specified

CHECKLIST FOR POST-OPERATIVE REPORT FOR NECK DISSECTION

CN8: Procedure and Specimen Site

☐ Fine needle aspiration biopsy (FNAB) - Sampled level of lymph nodes:

☐ Core biopsy - Sampled level of lymph nodes:

☐ Punch biopsy - Sampled level of lymph nodes:

☐ Incisional biopsy - Sampled level of lymph nodes:

☐ Excisional biopsy - Sampled level of lymph nodes:

☐ Radical neck dissection

(cervical lymph nodes [levels I-V],

☐ + sternocleidomastoid muscle,

☐ + internal jugular vein,

☐ +spinal accessory nerve,

☐ + submandibular salivary gland)

☐ Modified Radical (or Functional, or *Bocca*) Neck Dissection

(cervical lymph nodes [levels I-V],

☐ +/- sternocleidomastoid muscle

☐ +/- internal jugular vein

☐ +/- spinal accessory nerve,

☐ + submandibular salivary gland

CHECKLIST FOR POST-OPERATIVE REPORT FOR NECK DISSECTION

☐ Selective (or partial, or Regional) Neck Dissection (lymph node levels considered most likely to contain metastasis; specify):

- ☐ Level I (submental [IA], submandibular [IB]
- ☐ Level II (deep to *upper* third of the sternocleidomastoid muscle)
- ☐ Level III (deep to *middle* third of the sternocleidomastoid muscle)
- ☐ Level IV (deep to *lower* third of the sternocleidomastoid muscle)
- ☐ Level V (dorsal to the posterior border of the sternocleidomastoid muscle)
- ☐ Level VI (midline area of the neck extending from the hyoid bone to the suprasternal notch)
- ☐ Level VII (upper mediastinal lymph nodes)

☐ Extended Neck Dissection (additional lymph node levels and/or non-lymphatic structures)

- ☐ Additionally removed structure(s) (specify):

☐ Other (specify):

☐ Not specified

CN9: Specimen Orientation by the Surgeon to Aid Gross Pathology Understanding

☐ Annotations on a Schematic Diagram (*see attached*)

☐ Fixing Specimen to Cork or Plastic Plate and Marking Orientation by Pins or Sutures

☐ Labeling Lymph Node Groups and/or other Component Structures on Specimen

☐ Providing Specimens in Separate Containers Labelled According to their Locations

☐ Other (specify):

☐ Orientation Data Provided (at least 3 independent pieces of information to mark the 3 special axes; specify):

CHECKLIST FOR POST-OPERATIVE REPORT FOR NECK DISSECTION

☐ Special Markers (e.g. to indicate surgically critical margins, etc.; specify):

☐ None

Specimen Characteristics (PN1-2)

NOTE For PN1-2: select all that apply and identify with numbers if more than one specimen is received. Numbers must be consistent with those defined in CN7.

PN1: Specimen received

☐ Fresh

☐ In formalin

☐ In RNA Later

☐ As Cytologic Smear (number of slides received):

☐ Core Cylinder (number of cylinders received):

☐ Other (specify):

.

PN2: Specimen integrity

☐ Intact

☐ Fragmented

Specimen Gross Morphology (PN3-6)

NOTE for PN 3-6: multiply these sections and identify with numbers according to CN7 if more than one specimen is received. Select or fill out all that apply.

PN3: Specimen Size

Greatest Dimensions: ☐ x ☐ x ☐ cm

Additional Dimensions: ☐ x ☐ x ☐ cm
(if more than one part)

PN4: Orientational Information Received with Specimen

☐ Annotations on a Schematic Diagram (sent attached)

☐ Fixing Specimen to Cork or Plastic Plate and Marking Orientation by Pins or Sutures

☐ Labelling Lymph Node Groups and/or other Component Structures on Specimen

☐ Providing Specimens in Separate Containers Labeled According to their Locations

☐ Other (specify):

☐ Orientational Data provided (at least 3 independent pieces of information to mark the 3 special axes (specify):

☐ Special Markers (e.g. to indicate surgically critical margins, etc.; specify):

☐ None

PN5: Component Structures

☐ Submandibular Salivary Gland (specify dimensions): ☐ x ☐ x ☐ cm

☐ Sternocleidomastoid Muscle (specify dimensions): ☐ x ☐ x ☐ cm

☐ Node-containing Adipose Tissue (specify dimensions): ☐ x ☐ x ☐ cm

☐ External Jugular Vein (specify length): ☐ cm

☐ Internal Jugular Vein (specify length): ☐ cm

☐ Spinal Accessory Nerve

☐ Tail of Parotid Gland (specify dimensions): ☐ x ☐ x ☐ cm

CHECKLIST FOR POST-OPERATIVE REPORT FOR NECK DISSECTION

☐ Skin Flap (specify dimensions): ______ x ______ x ______ cm

☐ Other (specify):

Size of smallest and largest Lymph Nodes Identified (specify largest dimensions):

______ *Versus* ______ cm

Lymph Nodes Identified per Level:

Level I (submental [IA], submandibular [IB]

Total:

Grossly Metastatic:

Submitted Cassettes (Code of cassette [No. of lymph nodes; No of tissue pieces in cassette):

Level II (deep to ***upper third*** of the sternocleidomastoid muscle)

Total:

Grossly Metastatic:

Submitted Cassettes (Code of cassette [No. of lymph nodes; No of tissue pieces in cassette):

Level III (deep to ***middle third*** of the sternocleidomastoid muscle)

Total:

Grossly metastatic:

Submitted Cassettes (Code of cassette [No. of lymph nodes; No of tissue pieces in cassette):

Level IV (deep to ***lower third*** of sternocleidomastoid muscle)

Total:

Grossly Metastatic:

Submitted Cassettes (Code of cassette [No.of lymph nodes; No of tissue pieces in

cassette):

CHECKLIST FOR POST-OPERATIVE REPORT FOR NECK DISSECTION

Level V (dorsal to the posterior border of the sternocleidomastoid muscle)

Total: [ ]

Grossly Metastatic: [ ]

Submitted Cassettes (Code of cassette [No. of lymph nodes; No of tissue pieces in cassette):

[ ]

Level VI (midline area of the neck extending from the hyoid bone to the suprasternal notch)

Total: [ ]

Grossly Metastatic: [ ]

Submitted Cassettes (Code of cassette [No. of lymph nodes; No of tissue pieces in cassette):

[ ]

Level VII (upper mediastinal lymph nodes)

Total: [ ]

Grossly Metastatic: [ ]

Submitted Cassettes (Code of cassette [No. of lymph nodes; No of tissue pieces in

cassette): [ ]

☐ Gross size of Largest Lymph Node Metastasis (specify largest dimension): [ ] cm

☐ Obvious Metastatic Disease with Fusion of Lymph Nodes:

Level(s) of Lymph Nodes Involved by Mass: [ ]

Maximum Dimension: [ ] cm

Estimated Number of Lymph Nodes Involved in Mass: [ ] cm

☐ Tumor Extension to Sternocleidomastoid Muscle

☐ Tumor Extension to Adipose Tissue Independent of Lymph Nodes

☐ Grossly Evident Tumor Extension into External Jugular Vein

☐ Grossly Evident Tumor Extension into Internal Jugular Vein

☐ Tumor Extension to Submandibular Salivary Gland

☐ Tumor Extension to Tail of Parotid Gland

CHECKLIST FOR POST-OPERATIVE REPORT FOR NECK DISSECTION

☐ Tumor Extension to Skin

☐ Grossly No Metastatic Disease Evident

☐ Other (specify):

Metastatic Tumor Histomorphology (PN7-18)

NOTE for PN7: select all that apply.

PN7: Histologically Verified Tumor Extension

☐ Total Number of Lymph Nodes Identified under the Microscope:

☐ Lymph Nodes identified per Level:

Level I (submental [IA, submandibular [IB])

Total Examined:

Histologically Metastatic:

Extracapsular Tumor Extension:

☐ Not Identified

☐ Present (specify):

☐ Indeterminate

Level II (deep to *upper third* of the sternocleidomastoid muscle)

Total Examined:

Histologically Metastatic:

Extracapsular Tumor Extension:

☐ Not Identified

☐ Present (specify):

☐ Indeterminate

Level III (deep to *middle third* of the sternocleidomastoid muscle)

Total Examined:

Histologically Metastatic:

Extracapsular Tumor Extension:

☐ Not Identified

☐ Present (specify):

☐ Indeterminate

Level IV (deep to *lower third* of sternocleidomastoid muscle)

Total Examined:

Histologically Metastatic:

Extracapsular Tumor Extension:

☐ Not Identified

☐ Present (specify):

☐ Indeterminate

Level V (dorsal to the posterior border of the sternocleidomastoid muscle)

Total Examined:

Histologically Metastatic

Extracapsular Tumor Extension:

☐ Not Identified

☐ Present (specify):

☐ Indeterminate

CHECKLIST FOR POST-OPERATIVE REPORT FOR NECK DISSECTION

Level VI (midline area of the neck extending from the hyoid bone to the suprasternal notch)

Total Examined: __________

Histologically Metastatic: __________

Extracapsular Tumor Extension:

- ☐ Not Identified
- ☐ Present (specify): __________
- ☐ Indeterminate

Level VII (upper mediastinal lymph nodes)

Total Examined: __________

Histologically Metastatic: __________

Extracapsular Tumor Extension:

- ☐ Not Identified
- ☐ Present (specify): __________
- ☐ Indeterminate

☐ Histologically Measured Size of Largest Lymph Node Metastasis (specify largest dimension):

______ cm

☐ Tumor Extension to Sternocleidomastoid Muscle

☐ Tumor Extension to Adipose Tissue Independent of Lymph Nodes

CHECKLIST FOR POST-OPERATIVE REPORT FOR NECK DISSECTION

☐ Grossly Evident Tumor Extension into External Jugular Vein

☐ Grossly Evident Tumor Extension into Internal Jugular Vein

☐ Tumor Extension to Submandibular Salivary Gland

☐ Tumor Extension to Parotid Gland

☐ Tumor Extension to Skin

☐ No Metastatic Disease Evident Verified Histologically

☐ Other (specify):

<u>NOTE for PN8</u>: select all that apply and identify with numbers according to CN7 if more than one specimen is received. Any identification number may be allocated to more than one histologic type and, in turn, any histologic type may receive more than one identification number.

PN8: Histologic Type

☐ Squamous cell carcinoma, conventional

<u>Variants of Squamous cell carcinoma</u>

- ☐ Acantholytic squamous cell carcinoma
- ☐ Adenosquamous carcinoma
- ☐ Basaloid squamous cell carcinoma
- ☐ Carcinoma cuniculatum
- ☐ Papillary squamous cell carcinoma
- ☐ Spindle cell squamous carcinoma
- ☐ Verrucous carcinoma

NOTE for PN9: designate the most prevalent tumor beside all detectable grades when the metastatic tumor manifests more than one grade of differentiation

**PN9: Histologic Grade**	**Detectable grade(s)**	**Most prevalent**
☐ Not applicable		
☐ GX: Cannot be assessed		
☐ G1: Well differentiated		
☐ G2: Moderately differentiated		
☐ G3: Poorly differentiated		
☐ Other (specify):		

NOTE for PN10: select all that apply (if applicable).

PN10: TNM Descriptors

☐ m (multiple primary tumors)

☐ r (recurrent > not allowed into study)

☐ y (post-treatment)

NOTE for PN11-12: select one in each section.

PN11: Regional Lymph Nodes (pN)*

☐ pNX: Cannot be assessed

☐ pN0: No regional lymph node metastasis

☐ pN1: Metastasis in a single ipsilateral lymph node, 3 cm or less in greatest dimension

☐ pN2a: Metastasis in a single ipsilateral lymph node, more than 3 cm but not more than 6 cm in greatest dimension

☐ pN2b: Metastasis in multiple ipsilateral lymph nodes, none more than 6 cm in greatest dimension

☐ pN2c: Metastasis in bilateral or contralateral lymph nodes, none more than 6 cm in greatest dimension

CHECKLIST FOR POST-OPERATIVE REPORT FOR NECK DISSECTION

☐ pN3: Metastasis in a lymph node more than 6 cm in greatest dimension

Superior mediastinal lymph nodes are considered regional lymph nodes (level VII). Midline nodes are considered ipsilateral nodes.

PN12: Distant Metastasis (pM)

☐ Not Applicable

☐ pM1: Distant metastasis

Specify site(s), if known:

Source of pathologic metastatic specimen (specify):

<u>NOTE for PN13:</u> multiply this section and identify with numbers according to CN7 if more than one specimen is received. Within this section select all that apply.

PN13: Critical margin Indicated by the Surgeon with Special marker

☐ Cannot be assessed

☐ Margin Uninvolved

Distance from closest cancer part as measured under the microscope: ______ mm

Specify margin per orientation, if possible:

☐ Margin Involved

Specify margin per orientation, if possible:

☐ Not applicable

CHECKLIST FOR POST-OPERATIVE REPORT FOR NECK DISSECTION

NOTE for PN14-16: multiply these sections and identify with numbers according to CN7 if more than one specimen is received. If one or more of the changes represented in these sections are present, please specify the histologic subtype of squamous cell carcinoma involved.

PN14: Treatment Effect (applicable to carcinomas treated with neoadjuvant therapy)

☐ Not identified

☐ Present (e.g. keratin debris, necrosis, sign of organization or fibrosis, other; specify):

☐ Indeterminate

PN15: Lymph-Vascular Invasion

☐ Not identified

☐ Present (specify):

☐ Indeterminate

PN16: Perineural Invasion

☐ Not identified

☐ Present (specify):

☐ Indeterminate

CHECKLIST FOR POST-OPERATIVE REPORT FOR NECK DISSECTION

<u>NOTE for PN17-18:</u> select all that apply and identify with numbers according to CN7 if more than one specimen is affected.

PN17: Additional Pathologic Findings

☐ Specify:

☐ None identified

PN18: Ancillary Studies

Specify type(s):

Specify result(s):

Pathologist signature: ____________________ **Date:** ____________________
