# Supplementary material for: Neoadjuvant leukocyte interleukin injection immunotherapy improves overall survival in low-risk locally advanced head and neck squamous cell carcinoma –the IT-MATTERS study
Source: Pathol Oncol Res. 2025 Mar 21;31:1612084. doi: 10.3389/pore.2025.1612084 (PMC11968324; doi:10.3389/pore.2025.1612084)
Supplement: Supplementary file 2 [file DataSheet4.pdf]

CHECKLIST FOR PRE-MULTIKINE® TREATMENT FOR NECK LYMPH NODE SAMPLING

Patient ID: ☐ **Patient Initials** ☐

Case Report Sheet[1] Number: ☐

-Checklist for Pre-Neoadjuvant Report (abbreviated from the complete form to meet the needs of pre-neoadjuvant tumor diagnostics)-

Clinical Notifications (CN1-8)

Clinical Setting (CN1-6)

NOTE for CN 1-4: Select all that apply.

CN1: Clinical History

☐ Neoadjuvant Therapy

☐ Yes (specify type):

For Multikine treatment specify area (lymph node level) of drug instillation:

☐ Level I (submental [IA], submandibular [IB]

☐ Level II (deep to ***upper*** third of the sternocleidomastoid muscle)

☐ Level III (deep to the ***middle*** third of the sternocleidomastoid muscle)

☐ Level IV (deep to ***lower*** third of the sternocleidomastoid muscle)

☐ Level V (dorsal to the posterior border of the sternocleidomastoid muscle)

☐ Level VI (midline area of the neck extending from the hyoid bone to the suprasternal notch)

☐ Level VII (upper mediastinal lymph nodes)

☐ No

☐ Indeterminate

☐ Other (specify):

[1]A compilation by Glasz, T MD; 2nd Dept. of Pathology, Semmelweis University (Budapest, Hungary), based on relevant guidelines of the College of American Pathologists, The Royal College of Pathologists, AJCC/UICC TNM – 7th edition, as well as parts from Rosai and Ackerman's Surgical Pathology.

CHECKLIST FOR PRE-MULTIKINE® TREATMENT FOR NECK LYMPH NODE SAMPLING

CN2: Methods Performed for Metastatic Tumor Detection

☐ Visual Inspection

☐ Palpation

☐ Ultrasound

☐ Tomography (CT, MRI)

☐ Other (specify):

CN3: Metastatic Tumor Detection

☐ Evident on Visual Inspection

☐ Detectable on Palpation

☐ Detectable with Ultrasound

☐ Detectable with Tomography (CT, MRI)

☐ Metastatic disease is not evident with clinical detection methods

☐ Other (specify):

CN4: Metastatic Tumor Laterality

☐ Right

☐ Left

☐ Midline

☐ Not specified

☐ Uncertain

<u>NOTE for CN5:</u> Select one (1).

CN5: Metastatic Tumor Focality

☐ Single focus

CHECKLIST FOR PRE-MULTIKINE® TREATMENT FOR NECK LYMPH NODE SAMPLING

☐ Multifocal

<u>NOTE for CN6:</u> Select all that apply

CN6: Metastatic Tumor Site

☐ Level I (submental [IA], submandibular [IB]

☐ Level II (deep to the ***upper*** third of the sternocleidomastoid muscle)

☐ Level III (deep to the ***middle*** third of the sternocleidomastoid muscle)

☐ Level IV (deep to the ***lower*** third of the sternocleidomastoid muscle)

☐ Level V (dorsal to the posterior border of the sternocleidomastoid muscle)

☐ Level VI (midline area of the neck extending from the hyoid bone to the suprasternal notch)

☐ Level VII (upper mediastinal lymph nodes)

☐ Soft tissue of the neck (specify):

☐ Vessel involvement (specify):

☐ Skin involvement

☐ Other (specify):

☐ None detected

☐ Not specified

CHECKLIST FOR PRE-MULTIKINE® TREATMENT FOR NECK LYMPH NODE SAMPLING

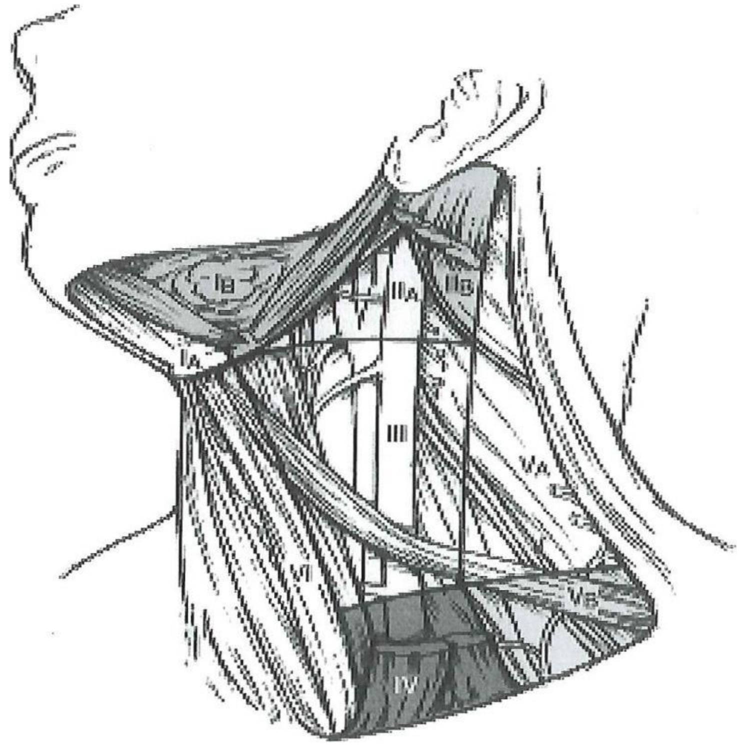

Tumor Sampling (CN7-8)

NOTE for sections CN7-8: select all that apply. Identify with numbers if more than one specimen is taken: in such a case any issue may receive more than one identification number. If numbers are given, they must be consistent here and in all subsequent sections of this Case Report Sheet with those defined in CN7.

CN7: Specimen Laterality

☐ Right

☐ Left

☐ Midline

☐ Not specified

CHECKLIST FOR PRE-MULTIKINE® TREATMENT FOR NECK LYMPH NODE SAMPLING

CN8: Procedure and Specimen Site

☐ Fine needle aspiration biopsy (FNAB)-Sampled level of lymph nodes:

☐ Core biopsy-Sampled level of lymph nodes:

☐ Punch biopsy-Sampled level of lymph nodes:

☐ Incisional biopsy-Sampled level of lymph nodes:

☐ Excisional biopsy-Sampled level of lymph nodes:

☐ Other (specify):

☐ Not specified

CHECKLIST FOR PRE-MULTIKINE® TREATMENT FOR NECK LYMPH NODE SAMPLING

Pathological Notifications (PN1-18)

Specimen characteristics (PN1-2)

<u>NOTES for PN1-2</u>: select all that apply and identify with numbers if more than one specimen is received. Numbers must be consistent with those defined in CN7.

PN1: Specimen Received

☐ Fresh

☐ In formalin

☐ In RNA later

☐ As Cytologic Smear (number of slides received):

☐ Core Cylinder (number of cylinders received):

☐ Other (specify): _

PN2: Specimen Integrity

☐ Intact

☐ Fragmented

Specimen Gross Morphology (PN3-6)

<u>NOTE for PN3:</u> multiply this section and identify with numbers according to CN7 if more than one specimen is received.

PN3: Specimen Size

☐ Greatest dimensions: ____ x ____ x ____ cm

Additional Dimensions (if more than one part):

☐ x ☐ x ☐ cm

Metastatic Tumor Histomorphology (PN7-18)

NOTE for PN7, select all that apply.

PN7: Histologically Verified Tumor Extension

☐ Lymph Nodes per Level

Level I (submental [IA, submandibular [IB])

Histologically Metastatic: ☐

Extracapsular Tumor Extension:

☐ Not Identified

☐ Present (specify): ☐

☐ Indeterminate

Level II (deep to the ***upper third*** of the sternocleidomastoid muscle)

Histologically Metastatic: ☐

Extracapsular Tumor Extension:

☐ Not Identified

☐ Present (specify): ☐

☐ Indeterminate

Level III (deep to *the* ***middle third*** of sternocleidomastoid muscle)

Histologically Metastatic: ☐

Extracapsular Tumor Extension:

☐ Not Identified

☐ Present (specify):

☐ Indeterminate

Level IV (deep to the ***lower third*** of the sternocleidomastoid muscle)

Histologically Metastatic:

Extracapsular Tumor Extension:

☐ Not Identified

☐ Present (specify):

☐ Indeterminate

Level V (dorsal to the posterior border of the sternocleidomastoid muscle)

Histologically Metastatic:

Extracapsular Tumor Extension:

☐ Not Identified

☐ Present (specify):

☐ Indeterminate

Level VI (midline area of the neck extending from the hyoid bone to the suprasternal notch)

Histologically Metastatic:

Extracapsular Tumor Extension:

☐ Not Identified

☐ Present (specify):

☐ Indeterminate

CHECKLIST FOR PRE-MULTIKINE® TREATMENT FOR NECK LYMPH NODE SAMPLING

Level VII (upper mediastinal lymph nodes)

Histologically Metastatic:

Extracapsular Tumor Extension:

☐ Not Identified

☐ Present (specify):

☐ Indeterminate

☐ Tumor Extension to Sternocleidomastoid Muscle

☐ Tumor Extension to Adipose Tissue Independent of Lymph Nodes

☐ Tumor Extension into External Jugular Vein

☐ Tumor Extension into Internal Jugular Vein

☐ Tumor Extension to Submandibular Salivary Gland

☐ Tumor Extension to Parotid Gland

☐ Tumor Extension to Skin

☐ No Metastatic Disease Evident Verified Histologically

☐ Other (specify):

NOTE for PN8, select all that apply and identify with numbers according to CN7 if more than one specimen is received. In such a case, any identification number may be allocated to more than one histologic type and, in turn, any histologic type may receive more than one identification number.

PN8: Histologic Type

☐ Squamous cell carcinoma, conventional

Variants of Squamous cell carcinoma

☐ Acantholytic squamous cell carcinoma

☐ Adenosquamous carcinoma

☐ Basaloid squamous cell carcinoma

CHECKLIST FOR PRE-MULTIKINE® TREATMENT FOR NECK LYMPH NODE SAMPLING

☐ Carcinoma cuniculatum

☐ Papillary squamous cell carcinoma

☐ Spindle cell squamous carcinoma

☐ Verrucous carcinoma

<u>NOTE:</u> For PN9, when the metastatic tumor manifests more than one grade of differentiation, please designate beside all detectable grades the most prevalent tumor grade, as well.

**PN9: Histologic Grade:**	**<u>Detectable grade(s)</u>**	**<u>Most prevalent</u>**
☐ Not applicable		
☐ GX: Cannot be assessed		
☐ G1: Well differentiated		
☐ G2: Moderately Differentiated		
☐ G3: Poorly differentiated		
☐ Other (specify):		

CHECKLIST FOR PRE-MULTIKINE® TREATMENT FOR NECK LYMPH NODE SAMPLING

<u>NOTE for PN15-16</u>:, multiply these sections and identify with numbers according to CN7 if more than one specimen is received. If one or more of the changes represented in these sections are present, please specify the histologic subtype of squamous cell carcinoma involved.

PN15: Lymph-Vascular Invasion

☐ Not identified

☐ Present (specify):

☐ Indeterminate

PN16: Perineural Invasion

☐ Not identified

☐ Present (specify):

☐ Indeterminate

<u>NOTE for PN 17-18</u>: select all that apply and identify with numbers according to CN7 if more than one specimen is affected.

PN17: Additional Pathological Findings

☐ Specify:

☐ None identified

PN18: Ancillary Studies

☐ Specify type(s):

CHECKLIST FOR PRE-MULTIKINE® TREATMENT FOR NECK LYMPH NODE SAMPLING

☐ Specify result(s):

Pathologist signature: ____________________

Date: ____________________
