# Supplementary material for: Neoadjuvant leukocyte interleukin injection immunotherapy improves overall survival in low-risk locally advanced head and neck squamous cell carcinoma –the IT-MATTERS study
Source: Pathol Oncol Res. 2025 Mar 21;31:1612084. doi: 10.3389/pore.2025.1612084 (PMC11968324; doi:10.3389/pore.2025.1612084)
Supplement: Supplementary file 3 [file DataSheet3.pdf]

CHECKLIST FOR POST-OPERATIVE PHASE PRIMARY TUMOR

Patient ID: ______ **Patient Initials** ______

Case Report Sheet[1] Number: ______

Clinical Notifications (CN1-7)

Clinical Setting (CN1-4)

NOTE: Select all that apply for CN1.

CN1: Clinical History

☐ Neoadjuvant Therapy:

- ☐ Yes (specify type):
- ☐ No
- ☐ Indeterminate

☐ Other (specify):

NOTE for CN2-3: select one in each section.

CN2: Tumor Laterality

☐ Right

☐ Left

☐ Bilateral

☐ Midline

☐ Not specified

CN3: Tumor Focality

☐ Single focus

☐ Multifocal (specify):

[1]A compilation by Glasz, T MD; 2nd Dept. of Pathology, Semmelweis University (Budapest, Hungary), based on relevant guidelines of the College of American Pathologists, The Royal College of Pathologists, AJCC/UICC TNM – 7th edition, as well as parts from Rosai and Ackerman's Surgical Pathology.

CHECKLIST FOR POST-OPERATIVE PHASE PRIMARY TUMOR

NOTE: Select all that apply for CN4.

CN4: Tumor Site

☐ Lateral border of tongue

☐ Ventral surface of tongue, NOS

☐ Dorsal surface of tongue, NOS

☐ Anterior two-thirds of tongue, NOS

☐ Upper gingiva (gum)

☐ Lower gingiva (gum)

☐ Anterior floor of mouth

☐ Floor of mouth, NOS

☐ Soft palate

☐ Buccal mucosa (inner cheek)

☐ Vestibule of mouth

- ☐ Upper
- ☐ Lower

☐ Alveolar process

- ☐ Upper
- ☐ Lower

☐ Mandible

☐ Maxilla

☐ Other (specify):

☐ Not specified

CHECKLIST FOR POST-OPERATIVE PHASE PRIMARY TUMOR

Tumor Sampling (CN5-7)

<u>NOTE for CN5-7</u>: select all that apply. Identify with numbers if more than one specimen is taken. If numbers are given, they must be consistent here and in all subsequent sections with those defined in CN5.

CN5: Specimen Laterality

☐ Right

☐ Left

☐ Midline

☐ Not specified

CN6: Procedure

☐ Fine needle aspiration biopsy (FNAB)

☐ Core biopsy

☐ Punch biopsy

☐ Incisional biopsy

☐ Excisional biopsy

☐ Resection

☐ Glossectomy (specify):

☐ Mandibulectomy (specify):

☐ Maxillectomy (specify):

☐ Palatectomy

☐ Neck (lymph node) dissection (specify):

CHECKLIST FOR POST-OPERATIVE PHASE PRIMARY TUMOR

☐ Other (specify):

☐ Not specified

CN7: Specimen Site

☐ Lateral border of tongue

☐ Ventral surface of tongue, NOS

☐ Dorsal surface of tongue, NOS

☐ Anterior two-thirds of tongue, NOS

☐ Upper gingiva (gum)

☐ Lower gingiva (gum)

☐ Anterior floor of mouth

☐ Floor of mouth

☐ Soft palate

☐ Buccal mucosa (inner cheek)

☐ Vestibule of mouth

- ☐ Upper
- ☐ Lower

☐ Alveolar process

- ☐ Upper
- ☐ Lower

☐ Mandible

☐ Maxilla

☐ Other (specify):

☐ Not specified

CHECKLIST FOR POST-OPERATIVE PHASE PRIMARY TUMOR

Pathological Notifications (PN1-21)

Specimen Characteristics (PN1-3)

NOTE for PN1-2: select all that apply and identify with numbers if more than one specimen is received. Numbers must be consistent with those defined in CN5.

PN1: Specimen Received

☐ Fresh

☐ In formalin

☐ In RNA later

☐ As cytologic smear (number of slides received):

☐ Other (specify):

PN2: Specimen Integrity

☐ Intact

☐ Fragmented

NOTE: Multiply section PN3 and identify with numbers according to CN5, if more than one specimen is received.

PN3: Specimen Size

Greatest dimensions: ___ x ___ x ___ cm

Additional dimensions (if more than one part):

___ x ___ x ___ cm

CHECKLIST FOR POST-OPERATIVE PHASE PRIMARY TUMOR

Tumor Gross Morphology (PN4-6)

NOTE for PN4: select all that apply and identify with numbers if more than one specimen is received. Numbers must be consistent with those defined in CN5.

PN4: Tumor Description

Gross subtype:

- ☐ Polypoid
- ☐ Exophytic
- ☐ Endophytic
- ☐ Ulcerated
- ☐ Sessile
- ☐ Other (specify):

NOTE for PN5-6: multiply the section and identify with numbers according to CN5 if more than one specimen is received.

PN5: Tumor Size

Greatest dimension: ____ cm

Additional Dimensions: ____ x ____ x ____ cm

☐ Cannot be determined (please comment):

PN6: Macroscopic Extent of Tumor

Specify:

CHECKLIST FOR POST-OPERATIVE PHASE PRIMARY TUMOR

Tumor Histomorphology (PN7-21)

NOTE for PN7-8: multiply each section and identify with numbers according to CN5 if more than one specimen is received.

PN7: Tumor Thickness (as defined histologically)

Tumor thickness: [ ] mm

Intact surface mucosa: [ ]

or ulcerated surface: [ ]

PN8: Microscopic Tumor Extension

Specify:

[ ]

NOTE for PN9: select all that apply and identify with numbers according to CN5 if more than one specimen is received. Any identification number may be allocated to more than one histologic type.

PN9: Histologic Type

☐ Squamous cell carcinoma, conventional

Variants of Squamous Cell Carcinoma

- ☐ Acantholytic squamous cell carcinoma
- ☐ Adenosquamous carcinoma
- ☐ Basaloid squamous cell carcinoma
- ☐ Carcinoma cuniculatum
- ☐ Papillary squamous cell carcinoma
- ☐ Spindle cell squamous carcinoma
- ☐ Verrucous carcinoma

NOTE: When tumor manifests more than one grade of differentiation, please designate both the highest and the most prevalent tumor grades for PN10.

CHECKLIST FOR POST-OPERATIVE PHASE PRIMARY TUMOR

**PN10: Histologic Grade**	**Detectable grade(s)**	**Most prevalent**
☐ Not applicable	☐	☐
☐ GX: Cannot be assessed	☐	☐
☐ G1: Well differentiated	☐	☐
☐ G2: Moderately Differentiated	☐	☐
☐ G3: Poorly differentiated	☐	☐

☐ Other (specify): ______

NOTE for PN11: select all that apply (if applicable).

PN11: TNM Descriptors

☐ **m (multiple primary tumors)**

☐ **r (recurrent > not allowed into study)**

☐ **y (post treatment)**

NOTE: For PN12-14, select one in each section.

PN12: Primary Tumor (pT)

☐ pTX: Cannot be assessed

☐ pT0: No evidence of primary tumor

☐ pTis: carcinoma in situ

☐ pT1: Tumor 2 cm or less in greatest dimension

☐ pT2: Tumor more than 2 cm but not more than 4 cm in greatest dimension

☐ pT3: Tumor more than 4 cm in greatest dimension

CHECKLIST FOR POST-OPERATIVE PHASE PRIMARY TUMOR

☐ pT4a: Moderately advanced local disease

Oral cavity: Tumor invades adjacent structures only (e.g. through cortical bone [mandible, maxilla], into deep [extrinsic] muscle of tongue [genioglossus, palatoglossus, and styloglossus], maxillary sinus, skin of face)

☐ pT4b: Very advanced local disease. Tumor invades masticator space, pterygoid plates, or skull base, and/or encases internal carotid artery

NOTE: Superficial erosion alone of bone/tooth socket by gingival primary is not sufficient to classify a tumor as T4.

PN13: Regional Lymph Nodes (pN)*

☐ pNX: Cannot be assessed

☐ pN0: No regional lymph node metastasis

☐ pN1: Metastasis in a single ipsilateral lymph node, 3 cm or less in greatest dimension

☐ pN2a: Metastasis in a single ipsilateral lymph node, more than 3 cm but not more than 6 cm in greatest dimension

☐ pN2b: Metastasis in multiple ipsilateral lymph nodes, none more than 6 cm in greatest dimension

☐ pN2c: Metastasis in bilateral or contralateral lymph nodes, none more than 6 cm in greatest dimension

☐ pN3: metastasis in a lymph node more than 6 cm in greatest dimension

Specify:

Number examined: ☐

Number involved: ☐

Size (greatest dimension) of the largest positive lymph node: ☐ cm

**Superior mediastinal lymph nodes are considered regional lymph nodes (level VII). Midline nodes are considered ipsilateral nodes.*

PN14: Distant Metastasis (pM)

☐ Not applicable

☐ pM1: distant metastasis

Specify site(s), if known:

☐

Source of pathologic metastatic specimen (specify):

☐

CHECKLIST FOR POST-OPERATIVE PHASE PRIMARY TUMOR

<u>NOTE:</u> Multiply section PN15 and identify with numbers according to CN5, if more than one specimen is received. Within this section select all that apply.

PN15: Margins (presence or absence of invasive cancer, carcinoma in situ or high grade [moderate or severe] dysplasia microscopically less than 5 mm from the surgical border)

☐ Cannot be assessed

☐ Margins uninvolved by invasive cancer, carcinoma in situ (including high grade [moderate or severe] dysplasia)

Distance from closest margin to either invasive or in situ carcinoma: ______ mm

Specify margin(s), per orientation, if possible:

☐ Margins involved by invasive carcinoma

Specify margin(s), per orientation, if possible:

☐ Margins involved by carcinoma in situ (includes moderate and severe dysplasia)

Specify margin(s), per orientation, if possible

☐ Not applicable

<u>Note for PN16-19</u>: multiply these sections and identify with numbers according to CN5 if more than one specimen is received. If one or more of the changes represented in these sections are present, please specify the histologic subtype of the squamous cell carcinoma involved.

CHECKLIST FOR POST-OPERATIVE PHASE PRIMARY TUMOR

PN16: Treatment Effect (applicable to carcinomas treated with neoadjuvant therapy)

☐ Not identified

☐ Present (specify):

☐ Indeterminate

PN17: Lymph-Vascular Invasion

☐ Not identified

☐ Present (specify):

☐ Indeterminate

PN18: Perineural Invasion

☐ Not identified

☐ Present (specify):

☐ Indeterminate

PN19: Lymph Nodes, Extranodal Extension

☐ Not identified

☐ Present (specify):

☐ Indeterminate

NOTE for PN20: select all that apply and identify with numbers according to CN5 if more than one specimen is affected,

PN20: Additional Pathologic Findings

CHECKLIST FOR POST-OPERATIVE PHASE PRIMARY TUMOR

☐ None identified

☐ Keratinizing dysplasia

- ☐ Mild
- ☐ Moderate
- ☐ Severe (carcinoma in situ)

☐ Non - keratinizing dysplasia

- ☐ Mild
- ☐ Moderate
- ☐ Severe (carcinoma in situ)

☐ Inflammation (specify type):

☐ Epithelial hyperplasia

☐ Colonization

- ☐ Fungal
- ☐ Bacterial

☐ Other (specify):

PN21: Ancillary Studies

☐ Specify type(s):

☐ Specify result(s):

Pathologist signature: __________________

Date: __________________
