# Supplementary material for: Neoadjuvant leukocyte interleukin injection immunotherapy improves overall survival in low-risk locally advanced head and neck squamous cell carcinoma –the IT-MATTERS study
Source: Pathol Oncol Res. 2025 Mar 21;31:1612084. doi: 10.3389/pore.2025.1612084 (PMC11968324; doi:10.3389/pore.2025.1612084)
Supplement: Supplementary file 4 [file DataSheet1.pdf]

Figure S1. Overall ITT

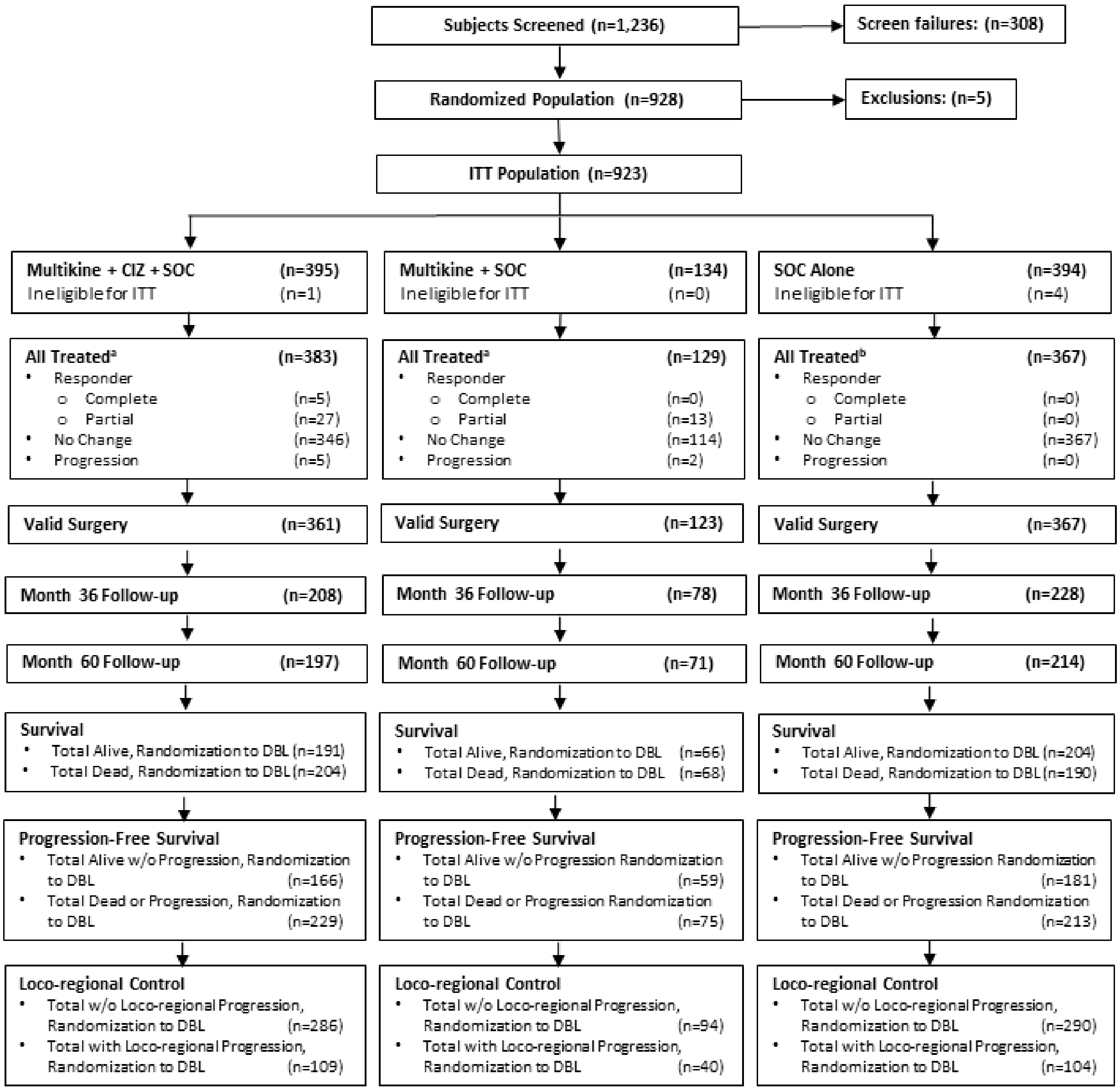

a: received LI(MK) regimen
b: no LI(MK) but underwent surgery

Abbreviations: CIZ=cyclophosphamide, indomethacin, zinc; DBL=database lock; ITT=intent-to-treat; Multikine=LI (Leukocyte Interleukin, Injection); SOC=standard of care.

Figure S2. Low risk ITT

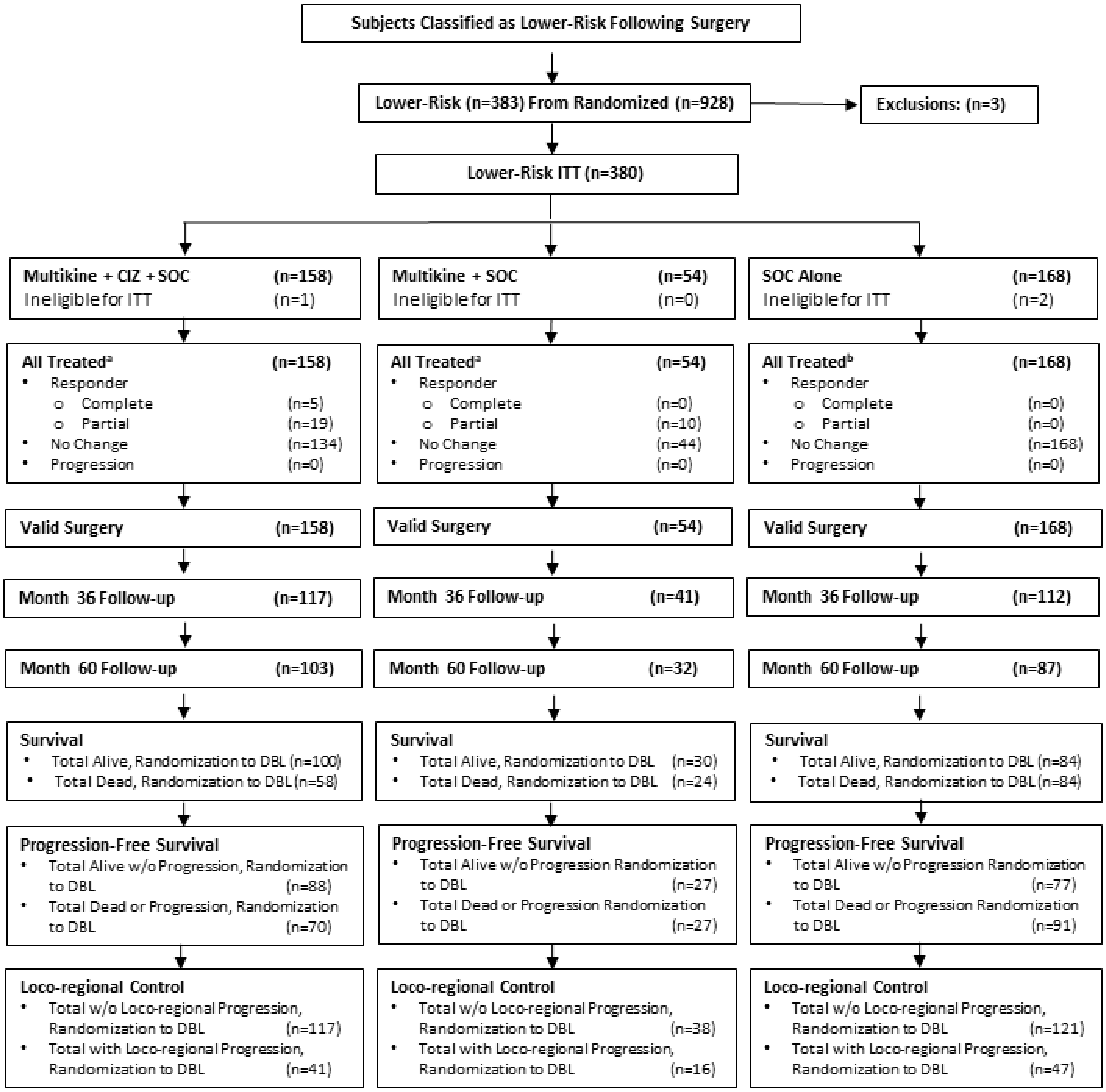

a: received LI(MK) regimen
b: no LI(MK) but underwent surgery

Abbreviations: CIZ=cyclophosphamide, indomethacin, zinc; DBL=database lock; ITT=intent-to-treat; Multikine=LI (Leukocyte Interleukin, Injection); SOC=standard of care.

Figure S3. High risk ITT

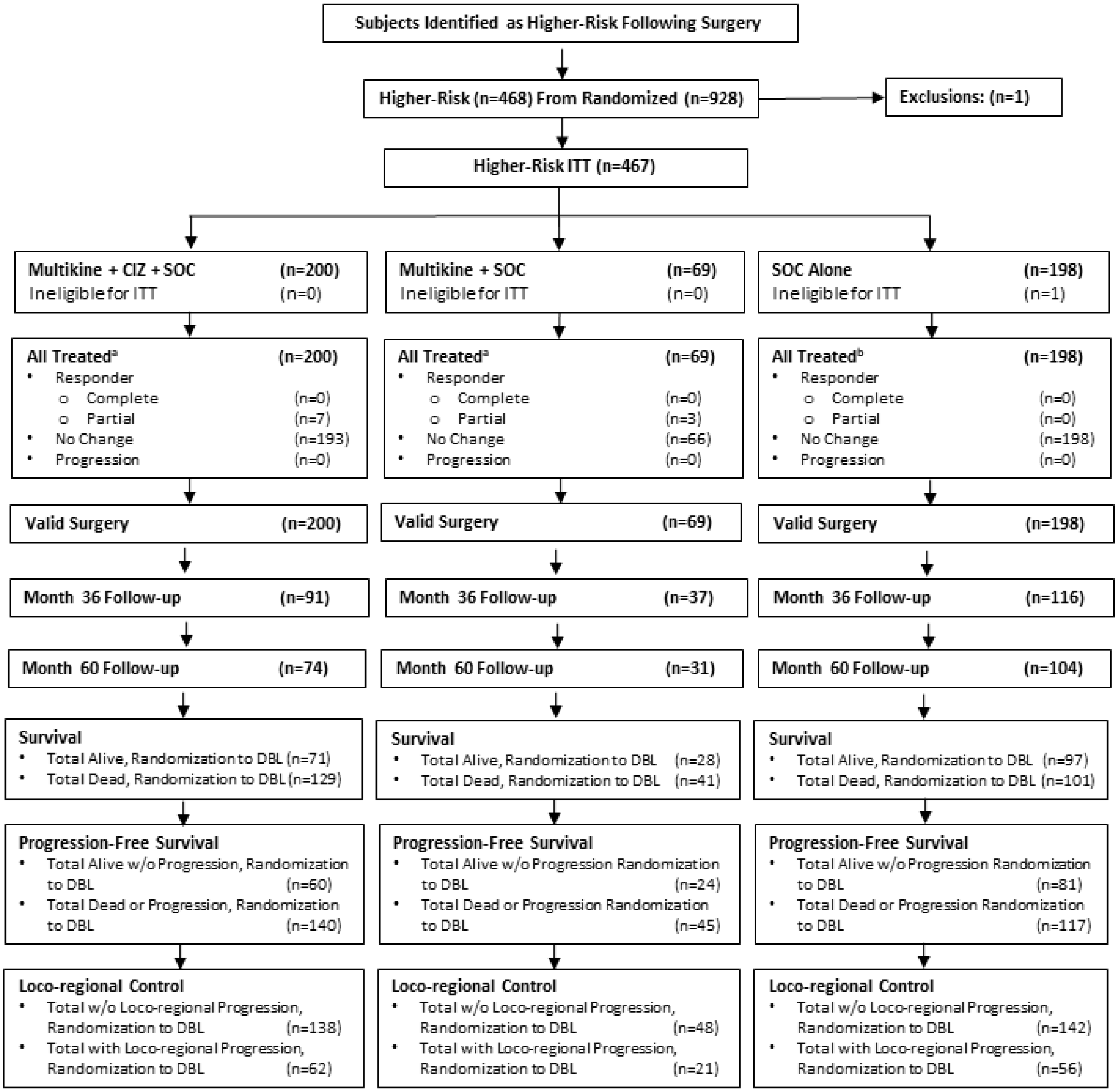

a: received LI(MK) regimen
b: no LI(MK) but underwent surgery

Abbreviations: CIZ=cyclophosphamide, indomethacin, zinc; DBL=database lock; ITT=intent-to-treat; Multikine=LI (Leukocyte Interleukin, Injection); SOC=standard of care.

Figure S4. Kaplan-Meier lifetable for OS in tumor stage III; low risk ITT population

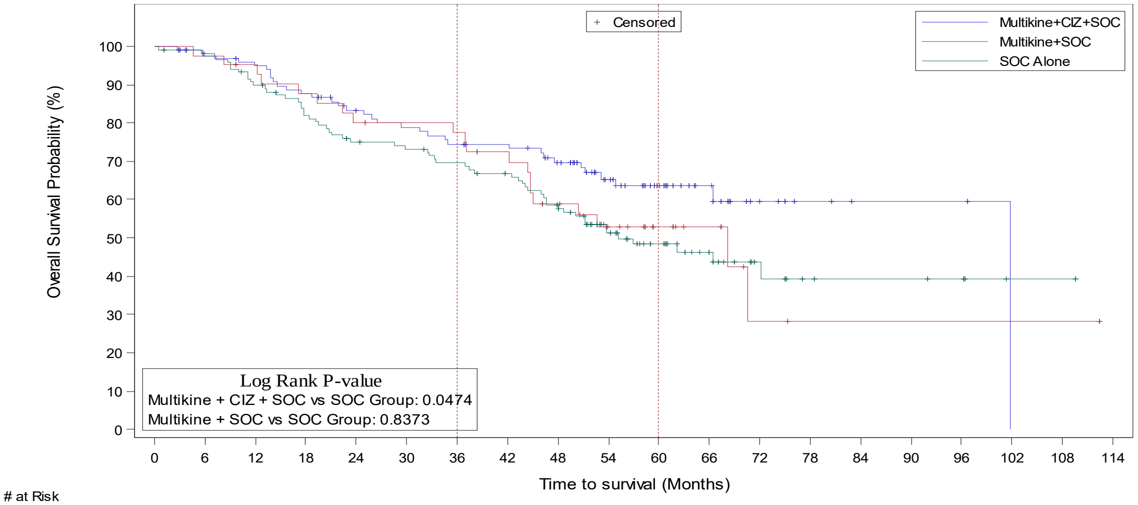

at Risk

Multikine+CIZ+SOC	102	95	91	84	75	71	67	65	57	39	27	17	8	4	2	2	2	0	0	0
Multikine+SOC	41	40	38	35	32	31	30	27	21	15	9	6	2	1	1	1	1	1	1	0
SOC Alone	119	115	105	94	85	82	77	72	61	42	28	19	10	6	5	5	4	1	1	0

Abbreviations: CIZ=cyclophosphamide, indomethacin, and zinc; ITT=intent-to-treat; OS=overall survival; SOC=standard of care. Multikine=LI (Leukocyte Interleukin, Injection).

Figure S5. Kaplan-Meier lifetable for OS in the RTx DDT low risk ITT population

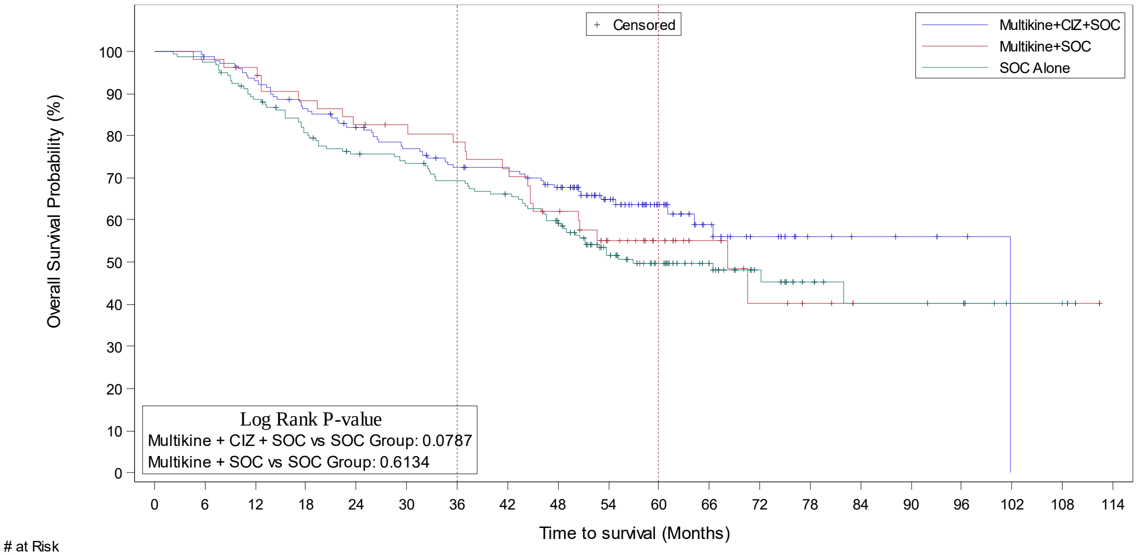

at Risk

	0	6	12	18	24	30	36	42	48	54	60	66	72	78	84	90	96	102	108	114
Multikine+CIZ+SOC	141	138	130	120	111	103	95	93	83	57	36	20	12	6	4	3	2	0	0	0
Multikine+SOC	53	52	50	45	42	40	38	35	29	21	14	9	5	3	1	1	1	1	1	0
SOC Alone	158	154	138	124	114	110	103	97	85	60	42	30	18	12	8	8	7	3	2	0

Abbreviations: CIZ=cyclophosphamide, indomethacin, and zinc; DDT=disease-directed therapy; ITT=intent-to-treat; OS=overall survival; RTx=radiotherapy; SOC=standard of care. Multikine=LI (Leukocyte Interleukin, Injection).

Figure S6. PFS Kaplan-Meier plot: ITT low risk patients in the 3 treatment groups (n=380)

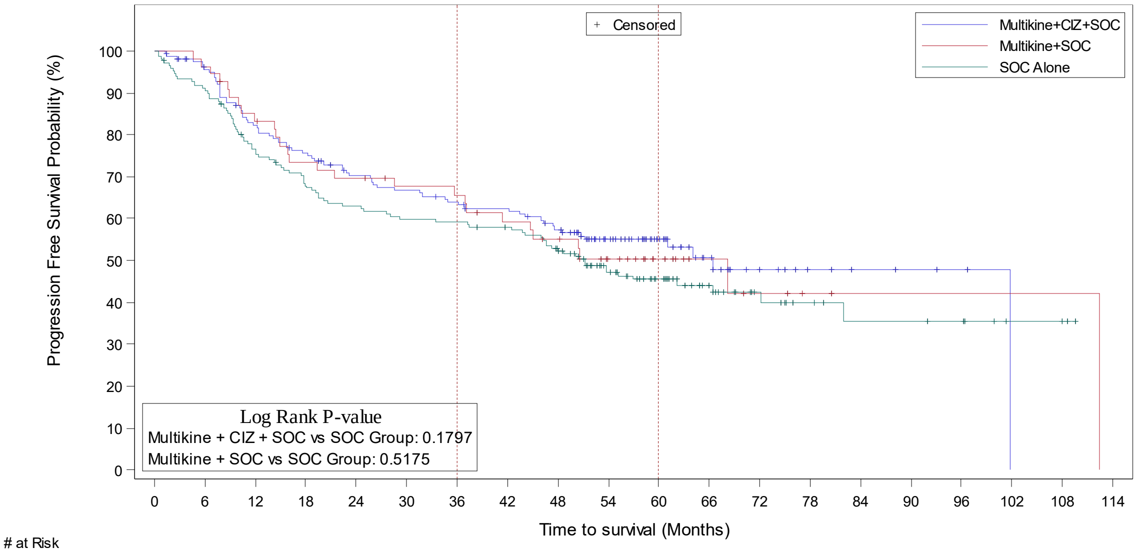

# at Risk	0	6	12	18	24	30	36	42	48	54	60	66	72	78	84	90	96	102	108	114
Multikine+CIZ+SOC	158	145	124	113	101	96	91	87	78	53	34	19	11	6	4	3	2	0	0	0
Multikine+SOC	54	52	44	38	36	33	32	28	25	18	11	6	4	2	1	1	1	1	1	0
SOC Alone	168	151	125	110	102	97	96	92	81	58	41	28	17	12	8	8	7	3	2	0

Abbreviations: CIZ=cyclophosphamide, indomethacin, zinc; ITT=intent-to-treat; PFS=progression-free survival; SOC=standard of care. Multikine=LI (Leukocyte Interleukin, Injection).

Figure S7. Locoregional control Kaplan-Meier plot: ITT low risk patients in the 3 treatment groups (n=380)

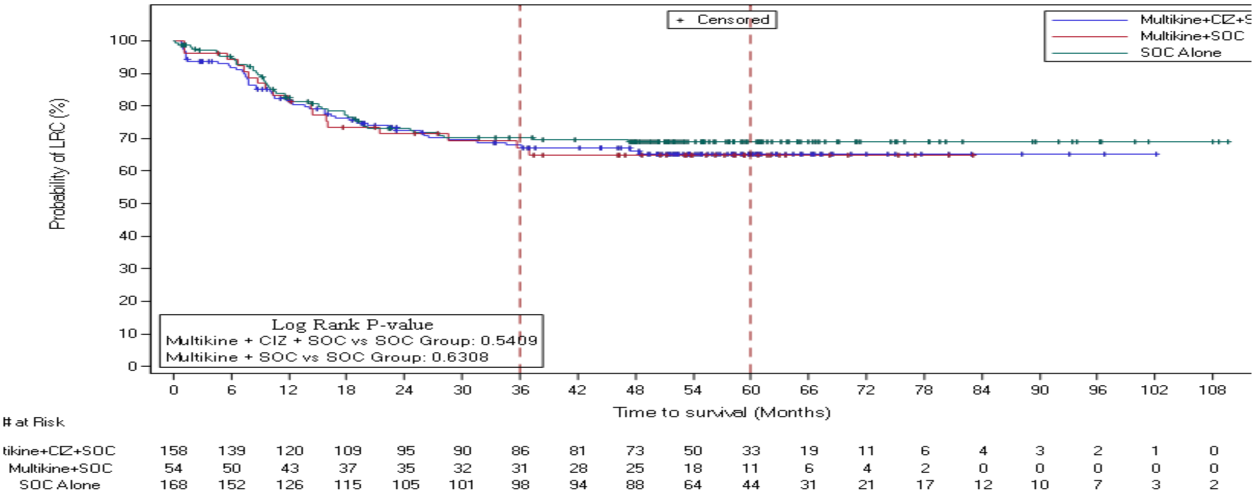

Abbreviations: CIZ=cyclophosphamide, indomethacin, zinc; ITT=intent-to-treat; SOC=standard of care. Multikine=LI (Leukocyte Interleukin, Injection).

Figure S8. Bar chart displaying TEAEs by study period – safety population (n=879)

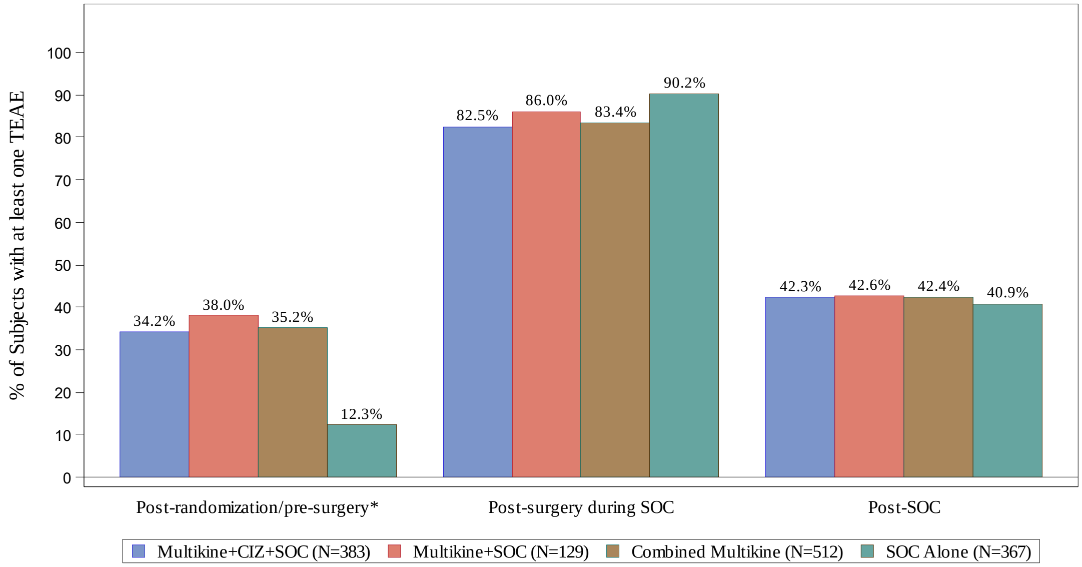

Abbreviations: CIZ=cyclophosphamide, indomethacin, and zinc as multivitamin supplement; SOC=standard of care; TEAE=treatment-emergent adverse event. Multikine=LI (Leukocyte Interleukin, Injection).

*Post-randomization/pre-surgery=From informed consent signature for all randomized subjects up-to surgery.

The post-randomization/pre-surgery interval is not adjusted for SOC (median 12 days) vs LI(MK) (median 35 days), thus requiring a 2.92 multiplier to adjust (resulting in a TEAE rate of 35.7%). All other intervals did not have time differences, thus not requiring adjustment.

Table S1. Demographics and clinical characteristics: overall ITT and low risk ITT

	ITT overall (N=923)			ITT low risk (N=380)		
	**Group 1**	**Group 2**	**Group 3**	**Group 1**	**Group 2**	**Group 3**
**Characteristic**	**(N=395)**	**(N=134)**	**(N=394)**	**(N=158)**	**(N=54)**	**(N=168)**
Mean age (years)	56.5	55.9	57.0	57.0	57.4	57.5
Male (%)	79.0%	78.4%	79.9%	77.8%	74.1%	83.3%
Caucasian (%)	78.7%	80.6%	80.5%	83.5%	85.2%	80.4%
Asian (%)	20.0%	18.7%	19.3%	15.8%	14.8%	19.6%
Not Hispanic or Latino (%)	48.1%	42.5%	47.2%	62.0%	53.7%	53.0%
Any alcohol consumption (%)	57.0%	59.0%	59.6%	52.5%	53.7%	58.9%
Any smoking history (%)	79.2%	82.8%	78.2%	72.2%	79.6%	79.8%
Any betel nut use history (%)	11.4%	11.9%	10.9%	8.9%	9.3%	11.3%
Asia-Pacific/Far-East (%)	5.3%	6.0%	5.1%	3.8%	3.7%	4.8%
Asia/West-Asia (%)	14.9%	14.2%	15.2%	10.8%	13.0%	14.9%
Europe (non-EU) (%)	24.1%	23.9%	24.6%	19.0%	16.7%	20.2%
Europe/Eurasia (%)	41.5%	41.8%	41.1%	53.8%	51.9%	48.8%
North America/Europe (EU) (%)	14.2%	14.2%	14.0%	12.7%	14.8%	11.3%
Mean BMI (kg/m^2)	24.30	24.03	24.16	25.06	24.89	24.62
Oral tongue (%)	46.1%	47.0%	45.2%	43.7%	44.4%	42.9%
Floor of mouth (%)	28.1%	27.6%	29.4%	31.6%	31.5%	33.3%
Cheek (buccal mucosa) (%)	13.4%	13.4%	14.0%	12.0%	13.0%	13.7%
Soft palate (%)	12.4%	11.9%	11.4%	12.7%	13.0%	10.1%
Surgery (%)	91.4%	91.8%	93.1%	100%	100%	100%
Low risk (%)	40%	40.3%	42.6%	100%	100%	100%
High risk (%)	50.6%	51.5%	50.3%	0%	0%	0%
Radiotherapy (%)	38.5%	46.3%	46.2%	89.2%	98.1%	94.0%
Chemoradiotherapy (%)	44.6%	39.6%	40.6%	2.5%	1.9%	2.4%
Positive surgical margin (%)#	16.7%	13.4%	15.2%	0%	0%	0.6%*
Extracapsular spread (%)	23.0%	24.6%	21.6%	0%	1.9%*	0.6%*

Abbreviations: BMI=body mass index; EU=European Union; ITT=intent-to-treat.
*See surgical findings section for an explanation.
#Includes both high and low risk – low risk had no positive margins (except one subject in SOC group)

Table S2. T, N, and AJCC stage at screening and surgery: overall ITT and low risk ITT

	ITT overall (N=923)			ITT low risk (N=380)		
	Group 1	Group 2	Group 3	Group 1	Group 2	Group 3
**Characteristic**	**(N=395)**	**(N=134)**	**(N=394)**	**(N=158)**	**(N=54)**	**(N=168)**
T stage at screening n (%)						
T1	21 (5.3)	4 (3.0)	12 (3.0)	12 (7.6)	1 (1.9)	6 (3.6)
T2	94 (23.8)	28 (20.9)	95 (24.1)	33 (20.9)	13 (24.1)	34 (20.2)
T3	163 (41.3)	68 (50.7)	191 (48.5)	70 (44.3)	32 (59.3)	89 (53.0)
T4a	117 (29.6)	34 (25.4)	96 (24.4)	43 (27.2)	8 (14.8)	39 (23.2)
T stage at surgery n (%)						
T0	3 (0.8)	0	0	3 (1.9)	0	0
T1	36 (10.0)	14 (11.5)	30 (8.2)	22 (14.0)	10 (18.5)	13 (7.7)
T2	111 (30.7)	26 (21.3)	118 (32.2)	46 (29.3)	13 (24.1)	48 (28.6)
T3	99 (27.4)	45 (36.9)	120 (32.7)	40 (25.5)	20 (37.0)	62 (36.9)
T4a	98 (27.1)	33 (27.0)	90 (24.5)	33 (21.0)	7 (13.0)	38 (22.6)
T4b	1 (0.3)	0	0	0	0	0
N stage at screening n (%)						
N0	190 (48.2)	62 (46.3)	180 (45.7)	98 (62.0)	32 (59.3)	108 (64.3)
N1	108 (27.4)	37 (27.6)	118 (29.9)	45 (28.5)	17 (31.5)	50 (29.8)
N2	96 (24.4)	35 (26.1)	96 (24.4)	15 (9.5)	5 (9.3)	10 (6.0)
N stage at surgery n (%)						
N0	132 (36.6)	51 (41.5)	118 (32.2)	102 (65.0)	39 (72.2)	101 (60.1)
N1	72 (19.9)	26 (21.1)	92 (25.1)	50 (31.8)	13 (24.1)	65 (38.7)
N2	149 (41.3)	42 (34.1)	151 (41.1)	3 (1.9)	1 (1.9)	1 (0.6)
N3	6 (1.7)	1 (0.8)	1 (0.3)	1 (0.6)	0	0
AJCC stage at screening n (%)						
III	218 (55.2)	75 (56.0)	228 (57.9)	102 (64.6)	41 (75.9)	119 (70.8)
IVa	177 (44.8)	59 (44.0)	166 (42.1)	56 (35.4)	13 (24.1)	49 (29.2)
AJCC stage at surgery n (%)						
III	123 (38.2)	44 (42.3)	152 (44.3)	89 (71.2)	31 (79.5)	113 (74.3)
IVa	199 (61.8)	60 (57.7)	191 (55.7)	36 (28.8)	8 (20.5)	39 (25.7)

Abbreviation: ITT=intent-to-treat.
Missing cases were those not undergone surgery or stage assessment

Table S3. TN stage at baseline by risk group (high risk and low risk) ITT population

TN at baseline	High risk (N=467)	Low risk (N=380)
T1N1	7	17
T1N2	8	2
T2N0	1	0
T2N1	60	64
T2N2	56	16
T3N0	116	164
T3N1	41	20
T3N2	51	7
T4AN0	48	74
T4AN1	29	11
T4AN2	50	5

Table S4. Multi-spectrum QoL metrics supporting CR and PR/CR benefit relative to NRs and to control (percent scoring best two ordinal scores per question)

Form question	Question description	Grp 1	Grp 2	Grp 3	CRs	PR/CR	NRs
EORTC11	Have you had trouble sleeping	85.5	86.8	88.6	**100.0***	**88.0**	85.4
EORTC12	Have you felt weak	85.2	89.4	89.0	**98.3**	**92.6**	85.1
EORTC13	Have you lacked appetite	88.8	89.7	88.4	**100.0**	91.8	88.5
EORTC18	Were you tired	86.5	88.5	89.6	**100.0**	91.8	86.1
EORTC19	Did pain interfere with activities	88.5	90.5	91.1	**100.0**	92.4	88.4
EORTC20	Difficulty concentrating on things	93.8	93.1	95.0	**100.0**	96.5	93.1
EORTC21	Did you feel tense	87.7	89.8	90.8	**100.0**	91.3	87.7
EORTC22	Did you worry	83.3	88.4	88.2	**100.0**	87.2	84.2
EORTC23	Did you feel irritable	88.5	93.6	90.9	**100.0**	93.5	89.2
EORTC24	Did you feel depressed	89.0	90.5	90.8	**100.0**	92.4	88.8
EORTC25	Difficulty remembering things	92.6	93.2	94.1	**100.0**	96.2	92.1
EORTC26	Phys. Cond. Interfered family life	87.8	88.5	90.8	**94.9**	92.9	87.1
EORTC27	Phys. Cond. Interfered social activ.	84.6	84.2	88.4	**98.3**	89.6	83.6
EORTC28	Phys. Cond. Cause financial dif.	77.2	77.5	79.1	**94.9**	85.3	75.9
EORTC30	Rate over. Qual. of life	34.3	41.2	39.3	**55.9**	**42.1**	35.0
EORTC31	Have you had pain in your mouth	86.7	82.4	87.5	**98.3**	**93.7**	84.1
EORTC32	Have you had pain in your jaw	89.0	87.6	88.6	**100.0**	**94.8**	87.5
EORTC33	Have you had mouth soreness?	82.6	82.8	83.2	**100.0**	88.5	81.6
EORTC34	Have you had a painful throat	92.0	91.7	93.5	**100.0**	96.4	91.1
EORTC35	Problems swallowing liquids	90.8	91.4	91.1	**100.0**	**97.3**	89.8
EORTC37	Problems swallowing solid food	67.3	71.1	67.6	**88.1**	**82.5**	65.7
EORTC43	Problems with your sense of smell	90.89	91.1	90.93	**100.0**	95.1	90.2
EORTC44	Problems with your sense of taste	85.5	84.1	84.9	**100.0**	89.3	84.4
EORTC46	Have you been hoarse	91.7	94.5	93.7	**100.0**	92.6	92.4
EORTC49	Trouble eating	80.1	82.4	81.5	**100.0**	**93.7**	78.4
EORTC52	Trouble enjoying your meals	82.2	79.8	82.7	**100.0**	89.1	80.2
EORTC53	Trouble talking to other people	83.0	81.4	85.1	**98.3**	92.1	80.9
EORTC54	Trouble talking on the telephone	83.1	83.2	84.1	**98.3**	92.6	81.4
EORTC55	Trouble social contact with family	94.0	94.2	94.5	**100.0**	99.2	93.1

Form question	Question description	Grp 1	Grp 2	Grp 3	CRs	PR/CR	NRs
EORTC56	Trouble soc. contact with friends	91.7	90.8	91.1	**100.0**	96.4	90.6
EORTC57	Trouble going out in public	90.5	87.4	89.2	**100.0**	95.6	88.7
EORTC58	Trouble phys. contact with family	94.0	93.2	94.4	**100.0**	97.5	93.1
EORTC61	Used pain killers	69.5	67.0	75.4	**93.2**	**79.5**	67.0
EORTC65	Gained weight	30.6	24.1	25.4	**44.1**	**30.4**	28.7
ALL 65	ALL 65 (including weight gain and loss)	84.1	84.9	85.4	**95.1**	**89.4**	83.4

Abbreviations: CR=complete response; NR=non-responders; PR=partial response; QoL=quality of life.
*Percent in Table – where 100.0 represents best score by respondents to the specific QoL question. Best score in ordinal scale with 100% being the maximal score=all respondents scored a response for the highest possible improvement from baseline.

Group 1 = LI+CIZ+SOC, Group 2 = LI+SOC, Group 3 = SOC only (Control)

Table S5. Overall summary of adverse events (excluding deaths, recurrences, and progressions) pre-surgery – safety population (n=879)

	Group 1	Group 2	Combined (Groups 1+2)	Group 3
	(N=383)	(N=129)	(N=512)	(N=367)
**Subject status**	**n (%)**	**n (%)**	**n (%)**	**n (%)**
Number of TEAEs	395	147	542	93
Number (%) of subjects with TEAEs	150 (39.2)	60 (46.5)	210 (41.0)	45 (12.3)
With any TEAE	150 (39.2)	60 (46.5)	210 (41.0)	45 (12.3)
With any related TEAE	65 (17.0)	25 (19.4)	90 (17.6)	0
With any LI-related TEAE	59 (15.4)	25(19.4)	84 (16.4)	NA
With any serious TEAE	3 (0.8)	1 (0.8)	4 (0.8)	2 (0.5)
With any serious related TEAE	1 (0.3)	1 (0.8)	2 (0.4)	0
With any serious LI-related TEAE	1 (0.3)	1 (0.8)	2 (0.4)	NA
With any CTC Grade 3 or 4 TEAE	9 (2.3)	2 (1.6)	11 (2.1)	2 (0.5)
With any TEAEs leading to death (excluding progression)	0	0	0	0
With any TEAE leading to discontinuation of study treatment	2 (0.5)	0	2 (0.4)	0
With any TEAE leading to discontinuation of LI	1 (0.3)	0	1 (0.2)	NA

Abbreviations: CTC=Common Toxicity Criteria; ITT=intent-to-treat; LI=Leukocyte, Interleukin, Injection; NA=not applicable; N=number of subjects in each group; n=number of subjects; TEAE=treatment-emergent adverse event.
Note: TEAE was any event that started after randomization or if present at randomization, worsened after randomization.

Group 1 = LI+CIZ+SOC, Group 2 = LI+SOC, Group 3 = SOC only (Control)

Note: CTC toxicity grade: 3=severe, 4=life-threatening.
Note: Related TEAEs were those events with a causality of ‘possible’, ‘probable’, `definitely’ or ‘related’.
Note: Group 3 did not receive any study directed treatment between entry and surgery.

Table S6. Specific TEAEs leading to death (subjects with any TEAEs leading to death – safety population)

	Group 1	Group 2	Combined (Groups 1+2)	Group 3
	(N=383)	(N=129)	(N=512)	(N=367)
	n (%)	n (%)	n (%)	n (%)
**Including deaths, recurrences, and progressions**				
Post randomization*	165 (43.1)	49 (38.0)	214 (41.8)	139 (37.9)
Pre-surgery	3 (0.8)**	1 (0.8)**	4 (0.8)**	0
During standard of care (DDT)	17 (4.4)	6 (4.7)	23 (4.5)	19 (5.2)
Post standard of care (DDT)	120 (31.3)	35 (27.1)	155 (30.3)	103 (28.1)
**Excluding deaths, recurrences, and progressions**				
Post randomization*	33 (8.6)	7 (5.4)	40 (7.8)	28 (7.6)
Pre-surgery	0	0	0	0
During standard of care (DDT)	6 (1.6)	1 (0.8)	7 (1.4)	6 (1.6)
Post standard of care (DDT)	18 (4.7)	2 (1.6)	20 (3.9)	15 (4.1)

Abbreviations: DDT=disease-directed therapy; N=number of subjects in each group; n=number of subjects; TEAE=treatment-emergent adverse event.

Group 1 = LI+CIZ+SOC, Group 2 = LI+SOC, Group 3 = SOC only (Control)

*Note: TEAEs start dates were used to define counts for pre-surgery, during SOC, and post-SOC while post-randomization includes all TEAE events after consent signature irrespective of TEAE start date. SOC did not receive LI (at any time in this study) and thus could not have LI-related TEAEs.

**All deaths occurred following DDT administration, but investigators declared pre-surgery start dates.
