# Supplementary material for: Neoadjuvant leukocyte interleukin injection immunotherapy improves overall survival in low-risk locally advanced head and neck squamous cell carcinoma –the IT-MATTERS study
Source: Pathol Oncol Res. 2025 Mar 21;31:1612084. doi: 10.3389/pore.2025.1612084 (PMC11968324; doi:10.3389/pore.2025.1612084)
Supplement: Supplementary file 5 [file DataSheet5.pdf]

CHECKLIST FOR PRE-MULTIKINE® TREATMENT FOR PRIMARY ORAL TUMOR

Patient ID: ______ **Patient Initials** ______

Case Report Sheet[1] Number: ______

Checklist for Pre-Neoadjuvant Report

(Abbreviated from the complete form to meet the needs of pre-neoadjuvant tumor diagnostics)

Clinical Notifications (CN1-7)

Clinical Setting (CN1-4)

NOTE for CN 1: Select all that apply

CN1: Clinical History

☐ Neoadjuvant Therapy

☐ Yes (specify type):

☐ No

☐ Indeterminate

☐ Other (specify):

NOTE for CN 2-3: In each of these sections, select one.

CN2: Tumor Laterality

☐ Right

☐ Left

☐ Bilateral

☐ Midline

☐ Not specified

[1]Modified by Glasz, T MD; 2nd Dept. of Pathology, Semmelweis University (Budapest, Hungary) on the basis of relevant guidelines of the College of American Pathologists, The Royal College of Pathologists, AJCC/UICC TNM – 7th edition.

CHECKLIST FOR PRE-MULTIKINE® TREATMENT FOR PRIMARY ORAL TUMOR

<u>NOTE for CN 3:</u> Select all that apply.

CN3: Tumor Focalilty

☐ Single focus

☐ Multifocal (specify):

NOTE for CN4: Select all that apply.

CN4: Tumor Site

☐ Lateral border of the tongue

☐ Ventral surface of the tongue, NOS

☐ Dorsal Surface of the tongue, NOS

☐ Anterior two-thirds of the tongue, NOS

☐ Upper gingiva (gum)

☐ Lower gingiva (gum)

☐ Anterior floor of the mouth

☐ Floor of the mouth, NOS

☐ Soft Palate

☐ Buccal mucosa (inner cheek)

☐ Vestibule of mouth

- ☐ Upper
- ☐ Lower

☐ Alveolar Process

- ☐ Upper
- ☐ Lower

☐ Mandible

☐ Maxilla

☐ Other (specify):

CHECKLIST FOR PRE-MULTIKINE® TREATMENT FOR PRIMARY ORAL TUMOR

☐ Not specified

Tumor Sampling (CN5-7)

NOTE for CN 5-7: In each section, select all that apply. Identify with numbers if more than one specimen is taken: in such a case, any issue may receive more than one identification number. If numbers are given, they must be consistent here and in all subsequent sections with those defined in CN5.

CN5: Specimen Laterality

☐ Right

☐ Left

☐ Midline

☐ Not Specified

CN6: Procedure

☐ Fine Needle Aspiration biopsy (FNAB)

☐ Core biopsy

☐ Punch biopsy

☐ Incisional biopsy

☐ Excisional Biopsy

☐ Resection

☐ Glossectomy (specify):

☐ Mandibulectomy (specify):

☐ Maxillectomy (specify):

☐ Palatectomy

☐ Neck (lymph node) dissection (specify):

CHECKLIST FOR PRE-MULTIKINE® TREATMENT FOR PRIMARY ORAL TUMOR

☐ Other (specify):

☐ Not specified

CN7: Specimen Site

☐ Lateral border of the tongue

☐ Ventral Surface of the tongue, NOS

☐ Dorsal surface of the tongue, NOS

☐ Anterior two-thirds of the tongue

☐ Upper gingiva (gum)

☐ Lower gingiva (gum)

☐ Anterior floor of mouth

☐ Floor of mouth, NOS

☐ Soft palate

☐ Buccal mucosa (inner cheek)

☐ Vestibule of mouth

- ☐ Upper
- ☐ Lower

☐ Alveolar process

- ☐ Upper
- ☐ Lower

☐ Mandible

CHECKLIST FOR PRE-MULTIKINE® TREATMENT FOR PRIMARY ORAL TUMOR

☐ Maxilla

☐ Other (specify):

☐ Not specified

Investigator signature: ____________________

Date: ____________________

CHECKLIST FOR PRE-MULTIKINE® TREATMENT FOR PRIMARY ORAL TUMOR

Pathological Notifications (PN1-21)

Specimen characteristics (PN1-3)

NOTE for PN 1-2: Select all that apply and identify with numbers if more than one specimen is received. Numbers must be consistent with those defined in **CN5**.

PN1: Specimen Received

☐ Fresh

☐ In formalin

☐ In RNA later

☐ As cytologic smear (number of slides received):

☐ Other (specify):

PN2: Specimen Integrity

☐ Intact

☐ Fragmented

NOTE for PN3: Multiply this section and identify with numbers according to CN5 if more than one specimen is received.

PN3: Specimen Size

Greatest dimensions: ___ x ___ x ___ cm

Additional dimensions (if more than one part): ___ x ___ x ___ cm

CHECKLIST FOR PRE-MULTIKINE® TREATMENT FOR PRIMARY ORAL TUMOR

Tumor Histomorphology (PN7-21)

NOTE for PN7-8: Multiply section and identify with numbers according to **CN5** if more than one specimen is received.

PN8: Microscopic Tumor Extension

Specify:

Note for PN9: Select all that apply and identify with numbers according to **CN5** if more than one specimen is received. Any identification number may be allocated to more than one histologic type.

PN9: Histologic Type

☐ Squamous cell carcinoma, conventional

Variant of Squamous Cell Carcinoma

☐ Acantholytic squamous cell carcinoma

☐ Adenosquamous carcinoma

☐ Basaloid squamous cell carcinoma

☐ Carcinoma cuniculatum

☐ Papillary squamous cell carcinoma

☐ Spindle cell squamous carcinoma

☐ Verrucous carcinoma

NOTE for PN10: When the tumor manifests more than one grade of differentiation, please designate both the highest and the most prevalent tumor grades.

**PN10: Histologic Grade**	**Highest**	**Most –prevalent**
☐ Not applicable		

CHECKLIST FOR PRE-MULTIKINE® TREATMENT FOR PRIMARY ORAL TUMOR

☐ GX: Cannot be assessed

☐ G1: Well differentiated

☐ G2: Moderately differentiated

☐ G3: Poorly differentiated

☐ Other (specify):

NOTE for PN16-19: Multiply these sections and identify with numbers according to **CN5** if more than one specimen is received. If one or more of the changes presented in these sections are present, please specify the histologic subtype of squamous cell carcinoma involved.

PN17: Lymph-Vascular Invasion

☐ Not identified

☐ Present (specify):

☐ Indeterminate

PN18: Perineural Invasion

☐ Not identified

☐ Present (specify):

CHECKLIST FOR PRE-MULTIKINE® TREATMENT FOR PRIMARY ORAL TUMOR

☐ Indeterminate

<u>NOTE for PN 20:</u> Select all that apply and identify with numbers according to **CN5** if more than one specimen is affected.

PN20: Additional Pathological Findings

☐ None identified

☐ Keratinizing dysplasia

☐ Mild

☐ Moderate

☐ Severe (carcinoma in situ)

☐ Non-keratinizing dysplasia

☐ Mild

☐ Moderate

☐ Severe (carcinoma in situ)

☐ Inflammation (specify type):

☐ Epithelial hyperplasia

☐ Colonization

☐ Fungal

☐ Bacterial

☐ Other (specify):

PN21: Ancillary Studies

CHECKLIST FOR PRE-MULTIKINE® TREATMENT FOR PRIMARY ORAL TUMOR

☐ Specify type(s):

☐ Specify result(s):

Pathologies signature: ____________________

Date: ____________________
